# G-quadruplex–R-loop interactions and the mechanism of anticancer G-quadruplex binders

**DOI:** 10.1093/nar/gkaa944

**Published:** 2020-11-02

**Authors:** Giulia Miglietta, Marco Russo, Giovanni Capranico

**Affiliations:** Department of Pharmacy and Biotechnology, Alma Mater Studiorum University of Bologna, via Selmi 3, 40126 Bologna, Italy; Department of Pharmacy and Biotechnology, Alma Mater Studiorum University of Bologna, via Selmi 3, 40126 Bologna, Italy; Department of Pharmacy and Biotechnology, Alma Mater Studiorum University of Bologna, via Selmi 3, 40126 Bologna, Italy

## Abstract

Genomic DNA and cellular RNAs can form a variety of non-B secondary structures, including G-quadruplex (G4) and R-loops. G4s are constituted by stacked guanine tetrads held together by Hoogsteen hydrogen bonds and can form at key regulatory sites of eukaryote genomes and transcripts, including gene promoters, untranslated exon regions and telomeres. R-loops are 3-stranded structures wherein the two strands of a DNA duplex are melted and one of them is annealed to an RNA. Specific G4 binders are intensively investigated to discover new effective anticancer drugs based on a common rationale, i.e.: the selective inhibition of oncogene expression or specific impairment of telomere maintenance. However, despite the high number of known G4 binders, such a selective molecular activity has not been fully established and several published data point to a different mode of action. We will review published data that address the close structural interplay between G4s and R-loops *in vitro* and *in vivo*, and how these interactions can have functional consequences in relation to G4 binder activity. We propose that R-loops can play a previously-underestimated role in G4 binder action, in relation to DNA damage induction, telomere maintenance, genome and epigenome instability and alterations of gene expression programs.

## INTRODUCTION

**Figure 1.**
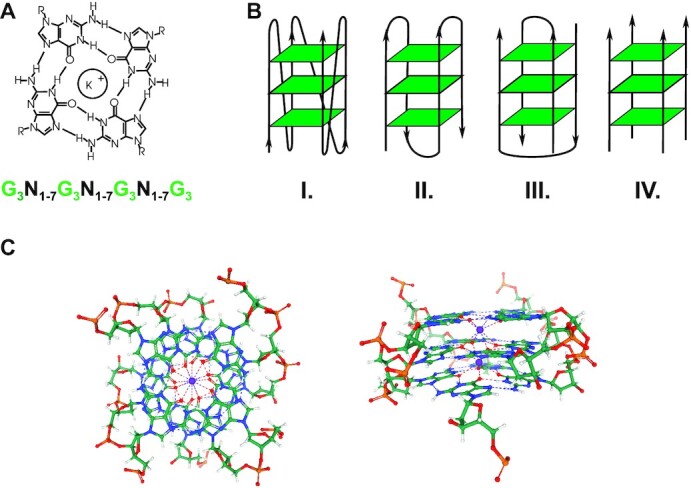
(**A**) The structure of a guanine quartet with a stabilizing K+ in the center channel. Below, the canonical potential G4-forming sequence (PQS). G, guanine; N, any nucleotide. (**B**) Different G4 structural conformations: (I) intra-strand parallel; (II) intra-strand anti-parallel; (III) inter-strand anti-parallel; (IV) inter-strand parallel. (**C**) NMR structure of a PQS present in the human KRAS proto-oncogene promoter (RCSB PDB ID: 5IV) (176).

The cell genome is constituted by B-form duplex DNA wrapped around histone octamers forming the highly conserved nucleosomal structure. Nevertheless, genomic DNA and cellular RNA can form a variety of non-B secondary structures, including G-quadruplex (G4), which can play major roles in the regulation of nucleic acid functions and genome stability in living cells. A G4 is formed by two or more stacked guanine tetrads held together by Hoogsteen hydrogen bonds and stabilized by K^+^ and Na^+^ (Figure 1). G4s are structurally polymorphic as guanines can come from the same or different strands (intra-strand and inter-strand G4s, respectively), different numbers of nucleotides can separate the tetrad guanines forming loops of different length and the strand orientation can be either parallel, antiparallel or a mix of them (Figure 1). Interestingly, G-rich sequences have been shown to adopt alternative conformational structures *in vitro* (1,2), raising the possibility that conformational changes of G4s are critical for regulation of cellular functions.

These structures can primarily form in G-rich stretches of the genome, such as in CpG islands, microsatellite and telomeric repeats, as well as in G-rich segments of RNAs (3). Convincing evidence primarily comes from genetic investigations of G4 functions (3,4), evolutionary conservation of potential G4-forming sequences (PQS) (5,6), the discovery of several G4-binding proteins in cells and viruses (3,4,7), NMR studies (8) and the visualization and genome mapping of G4s by chemical probes or specific antibodies (3,9). Bioinformatic tools have been developed to scan entirely prokaryotic and eukaryotic genomes to predict PQS (9,10). Even though the numbers of PQS can vary in a given genome, they are consistently enriched at key regulatory sites in eukaryotes, notably replication origins, gene promoters, untranslated exon regions, short sequence repeats and telomeres (11,12).

**Figure 2.**
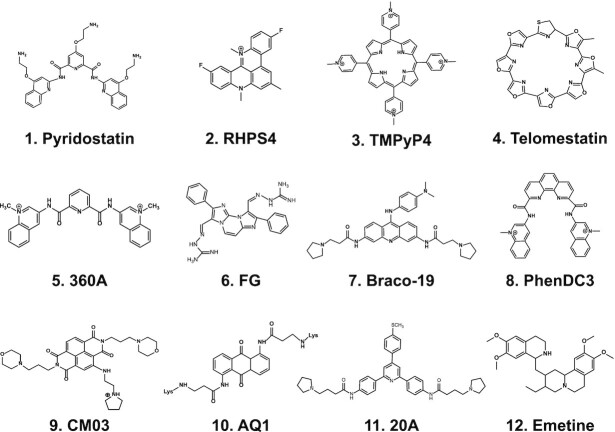
Chemical structures of G4 binders.

Research on G4 structures and functions is highly interrelated with an equally intense search for specific G4 binders endowed with therapeutic activity, in particular anticancer effects. Hundreds of compounds able to bind and stabilize G4s (Figure 2) have been developed with the aim to specifically target telomeric or oncogene promoter G4s in cancer cells (13–16). The general rationale was based on the observation that cancer cells can be addicted to activated driver oncogenes and/or to a proper regulation of telomeres to prevent senescence. Thus, specific downregulation of driver oncogenes or telomere destabilization could cause cell death or at least cell proliferation inhibition resulting in anticancer activity. Nevertheless, despite the high number of known G4 binders, such a selective molecular activity has not been definitely established in cancer models. In addition, certain G4 binders can interact with *i*-motifs (17) (a C-rich-strand non-canonical DNA structure (1)) raising questions about target specificity in living cells. More recent studies also showed that a nuclear enzyme, DNA Topoisomerase II, may be involved in the action of some G4 binders (18,19). Although Topoisomerase II-dependent molecular mechanisms remain to be fully defined, it is noteworthy that the enzyme may contribute to cell-killing activity of pyridostatin but not PhenDC3 (19), two structurally-different ligands (Figure 2). Thus, as few G4 binders have entered early phases of clinical trials and none has shown good efficacy in cancer patients yet (10–12), a deeper understanding of the mechanism of action of G4-interacting agents is needed to provide a strong rational for the development of ligands effective in cancer patients.

Here, we discuss published data that point to a different molecular action of G4 binders as these compounds may exert their biological activity through a more general mechanism rather than the inhibition of specific oncogenes or impairment of telomere maintenance. General mechanisms can be the induction of DNA damage and replication stress, an overall impairment of transcription and translation regulations, and the trigger of genomic and epigenetic instabilities. Such a mode of action should not be seen as a disadvantage in the discovery of effective anticancer G4 binders, as several FDA-approved drugs have pleiotropic effects at cellular and molecular levels, and still they get a specific pharmacological action in killing cancer cells. Here, we do not discuss important topics of G4 functions, enzymes resolving G4s and nanotechnological developments as they have been the subjects of recent excellent reviews (1–4,9–12,20–23).

### Lack of specific recognition of G4 structures by known ligands

Many of the known G4 binders have a planar aromatic moiety (Figure 2), which binds to G4 structures *via* π-π interactions with a terminal G-quartet (11,16). The ligand-G4 complex is further stabilized by electrostatic attraction between the G4 backbone phosphates and the protonated groups of the ligand. This type of non-specific molecular recognition allows an effective binding to a number of G4 targets, however most ligands do not discriminate among different conformational classes of G4 structures. Efforts have been reported to modulate the molecular recognition of small molecule towards a higher selectivity of G4-ligand interactions (14), however the design of compounds targeting only one or few G4 topologies or sequences is challenging as *in vivo* G4 polymorphisms are not fully determined and multiple G4 folds may be present at single sites in living cells.

Computational analyses of genomes have shown that PQS are widespread and non-randomly distributed in several different species (10,12). A feature that underlies the importance of G4s in biological processes is the high conservation of PQS in yeast species (5,6) and mammals (24–26). PQS are more conserved than expected by chance and nucleotides required to promote G4 formation are more conserved than surrounding nucleotides. More than 370 000 sequences have been predicted to form a G4 in the human genome (25,26). A recent study of genomic PQS showed that single-nucleotide-loop PQS (such as G3NG3NG3NG3) are most abundant in the genome of several species and are also conserved significantly in the human genome (6). The most conserved sequence in vertebrates (G3AG3AG3AG3) forms the *in vitro* least stable G4 and is the least prone to induce genomic instability in cells. Its frequency is higher than expected specifically in mammals (6). These findings show that most stable PQS are negatively selected in favour of least stable G4s to reduce their effects on genome stability while maintaining G4 structures with a biological function.

A different approach based on a polymerase-stop assay and Illumina sequencing was used to identify G4 structures in the human genome (27). The approach allowed the identification of PQS at sites of polymerase stops caused by G4 folding of the template DNA in the presence of pyridostatin (Figure 2). Interestingly, the authors found >700 000 PQS in the human genome, 63% of which was not predicted by bioinformatic analyses by Quadparser (24,25), including long-looped and bulged structures. The experimental mapping approach was recently improved and applied to identify PQS in 12 different species (28). Interestingly, PQS are enriched at control genic regions, such as promoters, in human and mouse genomes, but not in other species, suggesting a potential role in transcription regulation that seems to be specific for mammals and a few of other distant species (28). Moreover, the authors showed that pyridostatin stabilizes many different DNA G4 structures. In particular, G4s with only two guanine quartets were highly enriched in ligand-treated samples (28). Overall, pyridostatin can stabilize several different G4 structures in the human genome, including bulged, long-looped and two-quartet structures. Thus, the data indicate that a G4-interacting compound can bind and stabilize several different G4 assemblies, and this may likely result into low target selectivity in living cells.

### G4 formation: strand separation is necessary but likely not sufficient

G4 formation in living cells is under complex regulation mechanisms likely governed by protein factors and physical conditions of nucleic acids. The impact of helicases, endonucleases, polymerases and other specific factors on G4 regulation has been discussed recently (4,10,20,21). In living cells, a first constraint to G4 formation is the presence of chromatin structure, which likely poses a prerequisite for G4 formation: the removal of nucleosomes at PQS, as supported by the high G4 density observed at DNase I-hypersensitive sites (24,29). In addition, G4s have been mapped at nucleosome-free DNAs adjacent to fixed nucleosomes, suggesting a role of G4s in nucleosome positioning (30). Thus, a nucleosome at a PQS would counter G4 folding of the sequence, and repressive chromatin structures can prevent dangerous consequences such as G4-induced genomic instability (3,10).

Binding interactions of G4s with specific ligands have mostly been studied using single-stranded DNA substrates. However, with the exception of 5′-TTAGGG repeats at the 3′ of telomeres, G-rich sequences are usually paired with the complementary C-rich sequences throughout the genome. Many G4s can readily form in free single-stranded DNAs, nevertheless *in vitro* analyses showed that G-rich sequences prefer to form duplex rather than G4 under physiological conditions (31–34). Thus, with the exception of telomeres, the melting of a DNA duplex is the second prerequisite in living cells for the subsequent formation of G4s or other non-B structures. The energy for the melting of the two strands of a duplex can likely come from negative torsional tension generated by an elongating RNA polymerases, as predicted by the twin-supercoiling domain model (35). Behind the RNA polymerase, negative supercoils can be transformed into strand separation and this would allow the formation of G4s in G-rich stretches of melted strands. However, detailed analyses of G4 formation in relaxed and negatively-supercoiled plasmid DNAs have been reported showing that negative superhelicity is not sufficient to drive the formation of G4 in plasmids *in vitro* (30,36). Even though the local density of negative torsional tension may accumulate at higher levels in chromatin than in a plasmid *in vitro* (37), these findings are in contrast with results showing a ready formation of other non-B DNA structures (Z-DNA, cruciforms etc.) in negatively-supercoiled plasmids (38,39). The formation of non-B structures may however follow distinct mechanisms depending on the nature of the specific structure, in particular G4 assembly likely proceeds via a slower reaction constituted by discrete pre-folded intermediates (40). In fact, in comparison with other non-B secondary structures, G4 folding may be characterized by a higher kinetic barrier as the melting of a relatively longer duplex region is required for even the initial G-quartet formation (36).Therefore, negative torsional tension can be insufficient to drive G4 formation through strand separation.

In contrast with the above investigations, another study showed that a c-myc promoter G4 can readily form in negatively-supercoiled but not relaxed plasmids (41). At this specific promoter sequence, however, G4 assembly is coupled with the concomitant formation of an unusually stable i-motif on the opposite strand under physiological conditions (41). An i-motif is a non-canonical structure of a C-rich DNA strand containing C–C+ base pairs that requires slightly acidic conditions *in vitro* (1,11). Thus, the data document that the i-motif on one strand can stabilize the G4 on the other strand, which can explain the ready formation of G4s in a supercoiled plasmid (41). Interestingly, a different study reported that short peptide nucleic acids (PNA) able to bind a C-rich strand region of the human BCL2 promoter induced the formation of G4 in the opposite G-rich strand, and viceversa, the invasion of a PNA into the promoter DNA duplex required G4 formation (42). Collectively, these studies are therefore consistent with the hypothesis that the formation of a G4 in living cells requires both the negative torsional tension of the DNA duplex as well as a concomitant stabilization of the complementary strand, which may help in overcoming the kinetic barrier of G4 folding.

Contrasting results have been shown in the case of a microsatellite DNA found at about 350 bp from the insulin gene (insulin-linked polymorphic region) where either G4 or i-motif, but not both, can form in a linear duplex DNA under acidic conditions (43). The results may therefore depend on the experimental conditions that were not physiological in this case. However, non-B structures can compete with each other, regulating transcription as shown for the BCL2 gene (44,45). In nuclear chromatin, topological domains can dynamically change and supercoiling density is likely not uniform along a given genomic region (46). Nearby non-B structures can compete to absorb the mechanical energy stored in negative supercoils generated by the transcriptional apparatus (47). Thus, in living cells, the formation of any given G4 may also be dependent on the competing folding of nearby non-B structures, particularly in negatively-supercoiled DNA regions.

### Interplay among G4s, R-loops and DNA supercoiling

**Figure 3.**
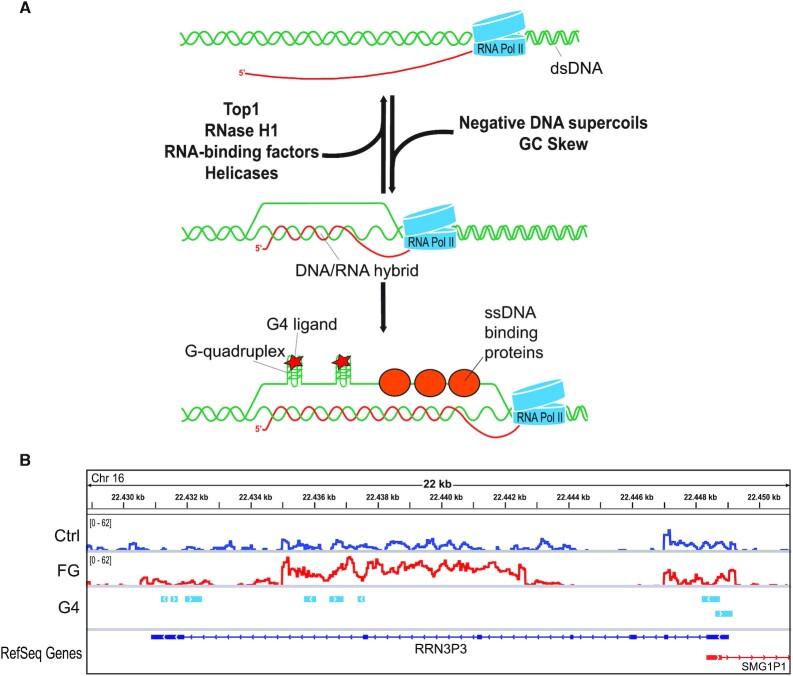
(**A**) Molecular model of the interplay among G4, R-loop, DNA supercoiling and protein factors. Top, main factors that contribute to prevent or promote R loop formation. Below, G4s and factors binding single-strand DNA can stabilize the displaced strand of R-loops. DNA and RNA are shown in green and red, respectively. (**B**) Genomic maps of G4-binder-induced R-loops and PQS established experimentally with a polymerase-stop assay (27) at the RRN3P3 gene locus. The graphs show normalized genomic R-loop profiles for control (blue line) and FG-treated (red line) U2OS cells (71) and oriented PQS (light blue boxes with white arrow).

A single-strand DNA in a promoter can be stabilized by several means, including binding to specific transcription factors (48), formation of a secondary structure (such as i-motif) or binding to a nucleic acid (DNA or RNA) other than the original complementary DNA strand. Among all the possibilities, the formation of a hybrid DNA:RNA duplex on the opposite strand can occur in case of R-loop, another non-canonical secondary structure. R-loops are three-strand structures wherein the two strands of a DNA duplex are melted and one of them is annealed to an RNA, forming a hybrid duplex, while the other strand is displaced out (Figure 3A). R-loops are favored by G-rich sequences on the coding strand as established by thermodynamic measures of the stability of DNA:DNA and DNA:RNA duplexes showing that the hybrid duplex is favored mainly in the presence of Gs in the RNA strand (49,50). Thus, in genomic regions with a G-rich coding strand, the formation of an R-loop would lead to a displaced strand that is rich in Gs therefore allowing the assembly of a G4 structure. It is worth considering that the length of PQS is in the order of few tens, whereas an R-loop can extend for hundreds or thousands of bases as shown in genome mapping studies (51,52) (see also Figure 3B). Therefore, interactions between the two structures do not likely involve the entire length of the displaced strand of R-loops.

As DNA:RNA hybrid formation also requires strand separation of the original DNA duplex, most important factors for R-loop formation are the negative torsional tension of the DNA duplex and the presence of a complementary RNA transcript, which naturally occurs in transcribed genes. The close structural relationship among negative DNA supercoiling, transcription and R-loops dates back to the 1970s of last century. As *in vitro* nascent transcripts were found in a nuclease-resistant complex with DNA depending on the superhelical density of the DNA template, Richardson (53) clarified that the nuclease-resistant transcript was bound to the template strand by base pairing forming a hybrid duplex, and that the formation of such hybrid is markedly affected by the superhelical tension of the studied bacteriophage PM2 DNA. This early observation established that *in vitro* transcription-dependent R-loop formation dramatically depends on negative supercoils of the template.

The relationships between R-loops and DNA topology, and the biological consequences of R-loop formation were then investigated in details in living cells by the rigorous work of M. Drolet and collaborators in the 1990s (54,55), anticipating the high and broad interests on R-loop biology of last two decades. Working with plasmids and *Escherichia coli*, they established that R-loops are most sensitive to the template DNA supercoiling level both *in vivo* and *in vitro*, and that altering the equilibrium balance of R-loop levels in the genome can lead to severe growth and segregation defects in bacteria (56,57). In agreement with this knowledge, bacterial DNA topoisomerase I, an enzyme able to reduce negative torsional tension of the DNA template, is a critical factor in modulating R-loop levels in *E. coli* cells (58,59).

More recent works have extended our knowledge on R-loop biology and DNA topology providing data showing that R-loops are a common non-canonical structure widely present in bacterial and eukaryotic nuclear and mitochondrial genomes (52,60–62). R-loops are dynamically formed at highly transcribed genes, in particular at their 5′ and 3′ ends. In mammalian cells, negative supercoiling can accumulate at transcription start sites of active genes thus favoring strand separation of the DNA template (30,37,63,64). Even though a low level of R-loops is present at any given locus at steady-state, however R-loops can cover 3–5% of the genome as determined in many organisms by several groups (65–71). In agreement with findings in bacterial cells, DNA topoisomerase I, which is very active in reducing negative supercoils, is a main player in modulating R-loop levels in nuclear chromatin of yeast (72), plants (73) and human cells (51). Consistently, human DNA topoisomerase I has been shown to be recruited along transcribed genes and is activated by the elongating RNA polymerase II to relax DNA supercoils generated by the enzyme, thus achieving an efficient transcription process (74). The impact of negative DNA torsional tension on DNA structure is not limited to R-loops and G4s, as dynamic topological changes can affect the formation of other non-canonical structures of the DNA template (30). Notably, the interplay between DNA supercoiling and non-canonical structures can have a critical function in regulating the transcription of the c-myc gene (37,46).

R-loop mapping along the genome provided information on R-loop length showing that they can extend for several hundreds of bases at each locus (64), therefore providing the melting of long sequences that is required for G4 formation on the opposite strand. An early demonstration that this can occur has come from N. Maizels’ laboratoy (75) working in *in vitro* systems as well as in bacterial cells. By using electron microscopy and biochemical assays, they showed that high transcription rates induced the formation of a novel structure termed G-loop, constituted by G4 structures on the G-rich non-template strand and a stable RNA/DNA hybrid on the template. Formation of G-loops was dependent on transcription, G-richness of the non-template strand, which can also reflect a higher stability of hybrid duplexes, and negative supercoiling of the template DNA. In agreement with these findings, a recent report showed that an intramolecular DNA G4 and a hybrid duplex promptly form upon *in vitro* transcription of a plasmid and, once formed, the two structures persist for several hours at physiological temperature even after transcription was stopped (76). Moreover, formation and persistence of the G-loop strongly depended on the hybrid duplex (76). Atomic force microscopy was used to analyze G-loops generated by transcription of a murine immunoglobulin Sc3 switch region cloned into a plasmid,showing that the structure was dependent on the presence of the hybrid duplexes as they disappear upon RNaseH1 treatment (77). Another study showed that the structural organization of the non-template strand is a fundamental feature of R-loops even though the structural characteristics of the non-template strand were not clearly defined (78). Interestingly, depletion of telomeric RNA transcripts (TERRA) in human cells resulted in a decrease in telomeric G4 structures suggesting that R-loops may affect G4 formation at telomeres (79). Overall, the findings are consistent with the hypothesis that the displaced strand of an R-loop can fold into G4 structures also in chromatin and that G4s and R-loops can likely assemble at the same time at highly active genes in living cells.

Interestingly, ssDNA-binding proteins have been reported to bind the displaced strand of R-loop. In particular, RPA (replication protein A), a heterotrimeric protein complex, can bind and stabilize ssDNA segments playing a critical role at replication forks to coordinate sensing of excess ssDNA, activation of DNA damage checkpoints, replication and recombination (80,81). RPA therefore can sense and stabilize the displaced strand of an R-loop and then recruit different factors to regulate R-loops during transcription preventing genome instability (82). Consistently, the ssDNA-binding protein of Arabidopsis AtNDX can also bind to the displaced strand and stabilize the R loop at the COOLAIR promoter resulting in the inhibition of COOLAIR transcription (83). Therefore, altogether the findings support that the formation of a G4 in one strand is highly favored by a hybrid duplex in the opposite strand, and viceversa, the formation of an R-loop is highly favored by the stabilization of the displaced strand by G4s or ssDNA-binding proteins (Figure 3A).

### Hybrid G4s and R-loops

G4 structures are heterogenous and many distinct conformations have been reported (1,2,11,84), however their roles in the mechanism of action of G4 binders is not known. A peculiar inter-strand G4 structure has been described in the conserved sequence block II (CSB II) of human mitochondrial genome (85,86). CSB II is a G-rich sequence that critically regulates the initiation of leading-strand replication starting from an RNA primer generated by the transcriptional apparatus. During transcription of CSB II, a long-lived R-loop can form likely due to the formation of a stable hybrid G4 constituted by the non-template DNA strand and the nascent RNA. The stability is likely due to the RNA, which is annealed to the DNA template with its 5′ portion while forming the hybrid G4 with its 3′ portion. The peculiar structure will lead to the stop of transcript synthesis therefore allowing the use of the RNA as a primer for DNA synthesis in human mitochondria. The hybrid G4 can therefore play a main role in transcription inhibition and regulation of mitochondrial DNA replication (85,86). A recent paper showed that RHPS4, a known G4 binder, is unable to induce DNA damage in the nucleus at low doses, however it can trigger mitochondrial respiratory-complex depletion by impairing specifically mitochondrial transcription (87). Although the paper did not identify the target of the RHPS4, the authors suggest a specific impairment of transcription regulation at the CSB II locus, raising the question of whether RHPS4 may specifically interact with the hybrid G4 structure involving the CSB II sequence.

The discovery of R-loop/hybrid G4 structure emphasizes the high conformational potential of non-B secondary structures and their specific functions. Hybrid G4s may also be present at many loci of the nuclear genome, as predicted by bioinformatic analyses, the *in vitro* detection of such a structure at the human NRAS promoter (88,89) and at human telomeres (90,91). Interestingly, hybrid G4s may also occur at the immunoglobulin heavy-chain (IgH) locus during class switch recombination (CSR) (92). The IgH locus has G-rich sequences at the switch region likely forming G4 structures, which can in turn bind and recruit the AID enzyme promoting DNA mutations (93). The switch region can also forms R-loops, which trigger the recombination mechanism leading to the change of the constant region of immunoglobulins (94). New findings showed that DEAD-box RNA helicase 1 (DDX1) is needed for CSR in murine B lymphocytes. Interestingly, DDX1 interacts with RNA G4s formed in the transcript from the switch region, promoting their resolution and the annealing of the same RNA sequence to the template DNA strand forming an R-loop over the switch region (92). Consistently, stabilization of the RNA G4 with an RNA-specific ligand prevented the formation of the R-loop. As the same PQS is present in both the transcript and the DNA, the authors proposed that hybrid DNA:RNA G4s might form at the IgH locus switch region involving the non-coding RNA and the displaced strand of the R-loop. The new DDX1-dependent mechanism therefore explains the recruitment of AID to the displaced strand of the R-loop, thus allowing the mutation of the DNA strand by AID (92,95). It is noteworthy that non-toxic doses of RHPS4 (Figure 2) have been reported to inhibit CSR in murine B cells in culture and in animals, showing a therapeutic effect on allergic inflammation (96).

Thus, the findings overall provide evidence of the occurrence in living cells of DNA:RNA hybrid G4s and their close interplay with R-loops. Interestingly, R-loop formation may be required to recruit RNA G4s at specific sites, such as the mitochondrial CSB-II motif and IgH locus switch region. Structures constituted by hybrid G4s and R-loops are exciting novel themes in the G4 field as they may offer more structural opportunities to design target-specific compounds.

### Dynamics of G4s and R-loops in human cells

The formation of G4s and R-loops can be visualized in cultured cells by immunofluorescence microscopy (IF) using the specific antibodies BG4 and S9.6. BG4 is a useful tools to detect G4s in living cells as it specifically recognizes intra-molecular and inter-molecular DNA and RNA G4 with high affinity (*K*_d_ = 0.5–2 nM) (97,98). S9.6 is a mouse monoclonal antibody developed by Bogluslawski *et al.* (99) that binds to DNA:RNA hybrids with nanomolar affinity. However, as it can also targets double-stranded RNA (100), appropriate controls are always needed in S9.6-based assays to detect R-loops in cells (51,64,71).

G4 foci were commonly observed after long time (24 h) of treatment with G4 binders (97), nevertheless, under these conditions it remains undetermined whether G4s were directly stabilized by the binder or indirectly mediated by cellular mechanisms. Recent kinetic analyses provided strong evidence that a number of structurally-unrelated G4 binders can stabilize G4 structures and increase the level of nuclear G4 foci in cultured cells at short time in a transient manner (71,101). G4 stabilization in cells follows a biphasic kinetic with an immediate (5 minutes) increase of G4 foci and a second phase of G4 level reduction, as detected by IF with the BG4 antibody (71). Cellular kinetics are very rapid as the number of G4 foci returns to initial levels in 20–30 min. The immediate induction of G4 foci is a proof that the studied compounds can act at their targets and stabilize G4 structures in nuclear chromatin of cancer cells.

Very similar kinetics were observed for R-loop levels in cells treated with G4 ligands (71) and with Topoisomerase I poisons (102,103). An immediate increase of R-loop levels, as detected with S9.6 antibody specific for DNA:RNA hybrids, followed by a marked decrease of R-loops. In the case of Topoisomerase I poisons, the biphasic kinetics paralleled the levels of Top1ccs (DNA-enzyme cleavage complexes, which are the molecular signature of poison activity), consistently with the hypothesis that poisoning of Top1 can directly cause the R-loop increase (102). Immediate molecular perturbations by topoisomerase I poisons at cellular levels are not restricted to R-loop levels and have been discussed previously (104).

Both G4s and R-loops can form at transcribed genes in living cells (52,60), nevertheless they are highly dynamic as several helicases, RNA-binding factors, endonucleases and DNA topoisomerases are active in cells to dissolve the structures restoring B-DNA duplexes and nucleosomes. The mechanisms of the biphasic curves remain to be established, however a simple hypothesis is that helicases or other enzyme able to dissolve G4/R-loop structures are promptly activated to respond to the raise of the structures. A steady-state equilibrium is generally set at low levels in cells and is likely a balanced outcome of molecular activities that induce the formation of G4s and R-loops, on one hand, and factors promoting their dissolution, on the other. Thus, the global levels of G4s and R loops are perturbed in a dynamic manner by external agents and cellular regulatory mechanisms promptly respond to restore the initial overall levels. Such dynamical R-loops / G4s interplay can have significant biological consequences as discussed for replication and DNA repair in a recent review (105).

### G-loop role in the induction of DNA damage by G4 binders

It is now established that chemical G4 stabilization can trigger genome-wide DNA double-stranded breaks (DSB) and genome instability, nevertheless the mechanisms of damage is not yet fully clarified, even though different molecular pathways leading to DSB formation have been proposed. One mechanism can be the direct cleavage of DNA at G4 structures, which may occur in some circumstances. G4a are recognized by several protein factors but are generally resistant to nucleases without a prior resolution of the secondary structure. Interestingly, the multifaceted DNA2 enzyme, critical for telomere stability, has been shown to have such an activity. DNA2 can bind and unwind telomeric G4s *in vitro*. In addition, its nuclease activity is activated by interactions with RPA, which is a determinant factor for specific G4 cleavage by DNA2 (106). The nuclease has been proposed to function during telomere replication and processing of Okazaki fragment at replication forks (106,107), possibly explaining G4-mediated DNA cleavage at telomeres and elsewhere in the genome during S phase. Interestingly, DNA2 depletion was shown to increase fragile telomeres induced by two G4 binders, TMPyP4 and telomestatin (Figure 2) (107), however, the role of DNA2 and other endonucleases in the induction of DNA cleavage by G4 binders needs to be established in relation to basic nuclear processes and genomic regions.

An important mechanism of DNA damage production can be the replication fork stalling and collapse occurring at a G4 structure on the template strand. G4 structures can constitute a physical impediment to replication progression at leading and lagging strands (3,21), and unresolved replication barriers trigger recombination-dependent restart of DNA synthesis and DSB formation likely by structure-specific endonucleases (for instance, MUS81 and XPF), active at yet undefined replication intermediates (23). DSB are usually investigated by determining the formation of γH2AX and p53BP1 foci, DSB molecular markers, and by assessing the ligand cellular effects such as the cell cycle arrest at G2/M phase and the activation of the DNA damage response pathway (DDR). The induction of replication-dependent DNA damage by G4 binders was firstly established at telomeres using different ligands causing a fragile telomere phenotype (108–110). However, it was soon established that G4 binders can cause DNA damage across the entire genome in cultured cells showing a high dependence on S-phase and DNA replication (111). G4 binders may have a stronger effect in cells deficient for the homologous recombination DSB repair (HRR) pathway, supporting a main role of the mechanism in G4 binder-induced DSBs. The knowledge came from observations in cancer cells deficient for BRCA1/2 genes, critical players of HRR, as G4 binders were shown to be more active in DSB accumulation and persistent checkpoint activation in these cell types (112). In addition, G4 binders were also more active in reducing cell proliferation and inducing chromosomal aberrations. As BRCA1/2 gene mutations have prognostic value in cancer patients, the findings can have a high impact for the development of G4-interacting compounds effective in clinical settings.

DNA breaks caused by a replication-blocking G4 have been investigated in the model organism C. elegans, revealing peculiar effects of G4 stabilization and a main error-prone repair mechanism leading to genetic instability (113). Genetic evidence showed that a persistent G4 structure can be transmitted through multiple mitotic divisions to daughter cells. As the persistent G4 can block replication causing a strand break gap in the annealed strand, the structure then leads into a DSB at the next round of DNA duplication in the daughter cell. The DSB is then repaired by an error-prone mechanism based on DNA polymerase theta (POLQ) leading to specific DNA deletions (113). Interestingly, translesion synthesis DNA polymerases were found to protect C. elegans from genomic deletions caused by G4-induced replication blocks (114). As molecular and genetic differences exist between *Caenorhabditis elegans* and mammalian cells, these mechanisms remain to be defined in human cells.

Another important mechanism of G4 binder-induced DNA damage is more related to the transcription process, as transcribing PQS can be challenging and can lead to genome instability. This mechanism has been suggested by investigating DNA damage caused by pyridostatin (Figure 2). Treatment of cancer cells with the ligand resulted in a fraction of γH2AX foci that was transcription dependent, and DSBs induced by pyridostatin were mapped at transcribed genes enriched for PQS, such as ribosomal genes and the SRC oncogene, but not at inactive genomic regions (111). Some insights into the transcription-dependent mechanism was reported recently. Stabilization of G4s by three structurally unrelated ligands (pyridostatin, FG and Braco-19, Figure 2) in cancer cells was shown to increase the global levels of nuclear G4/R-loop structures (71). The genome-wide mapping of R-loops in cells treated with either one of two binders (pyridostatin or FG) showed that G4 binder-induced R-loop peaks were commonly found in transcribed genes and were longer than corresponding peaks in untreated cells (Figure 3B) (71). The findings supported a simple mechanistic model in which binder-stabilized G4s in the displaced strand of an R-loop can result in an overall stabilization of G4/R-loop structures with a longer hybrid duplex at transcribed genes (Figure 3), in agreement with transcription-dependent G-loop formation in E. coli and plasmids (75). These findings were recently confirmed with monohydrazone derivatives that specifically bind to G4s (101).

**Figure 4.**
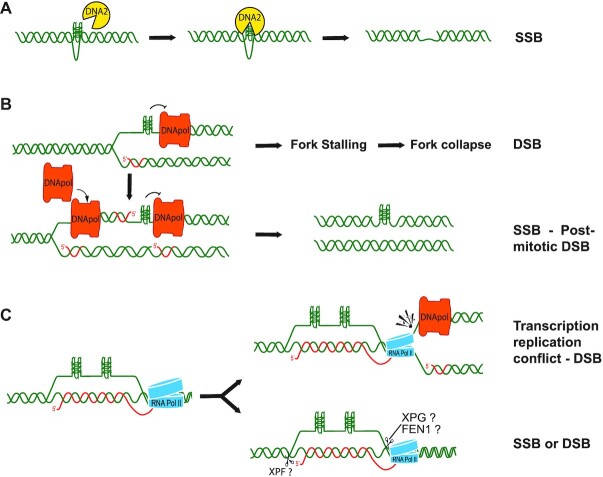
Mechanisms of DNA breakage formation induced by G4 stabilization. (**A**) G4 can be bound by a nuclease (DNA2) that then cleaves the DNA strand. (**B**) G4s can be a barrier to DNA synthesis by DNA polymerases leading to either fork collapse and DNA damage or re-priming by a second DNA polymerase downstream to the G4 generating a single-strand break (SSB). The SSB then becomes a double-strand break (DSB) at the next round of DNA replication in daughter cells. (**C**) G4s can stabilize co-transcriptional R-loops which can cause replication/transcription conflicts (top) or activate a repair pathway generating DNA breaks (below). DNA and RNA are shown in green and red, respectively.

Interestingly, G4/R-loop increase preceded the formation of DNA damage, as shown by formation of γH2AX foci, and overexpression of RNaseH1 in cells abolished DNA damage and cell death induced by the studied G4 binders (71). Moreover, G4 binders caused the generation of micronuclei at later times of cell growth in an R-loop-dependent manner, and BRCA2 silencing enhanced the overall effect (71). Micronuclei are chromatin fragments enveloped by a nuclear double-layer membrane and are generated following cell division defects including mis-segregation of chromosomes or chromosome portions. As micronuclei constitute a clear marker of genome instability, these recent findings show that G4 binders can increase genome instability in cancer cells through a mechanism dependent on R-loops and recombination repair (Figure 4). Collectively, the findings thus support a role for G-loops in the induction of DNA cleavage and micronuclei by G4 binders. The mechanism of DSB formation is unknown, however R-loops can be substrate of structure-specific endonucleases such as XPF and XPG, which are main players of transcription-coupled repair pathways (115–118). The cleavage of both DNA strands by XPF/XPG at the boundary of the hybrid may then lead to DSB (Figure 4), however whether these or other nucleases (117) can process G-loops remain to be established (Table 1).

**Table 1.** R-loop roles in molecular mechanisms of G4 binder activity in cancer cells

	G4 binder	Molecular or biological effects	R-loop role	Reference
1	Pyridostatin	DNA damage and micronuclei formation,	Yes	(71)
		Impairment of epigenetic memory	Possible	(143,144)
2	RHPS4	Mitochondrial transcription interference through binding to DNA:RNA hybrid G4	Possible	(85,87)
3	TMPyP4	Telomeric G4 stabilization	Possible	(107,138)
		DNA2 interference	Unknown	
4	Telomestatin	Telomeric G4 stabilization	Possible	(107)
		DNA2 interference	Unknown	
5	360A	Replication-dependent DNA damage at telomeres	Possible	(110)
6	FG	DNA damage and micronuclei formation,	Yes	(71)
		Cancer cell killing	Yes	(71)
7	Braco-19	DNA damage,	Yes	(71)
		G4 transcriptomic landscape changes	No	(172)
8	PhenDC3	G4 transcriptomic landscape changes	No	(172)
		Impairment of epigenetic memory	Possible	(143,144)
9	CM03	Global changes of gene expression	Possible	(151)
10	AQ1	Global changes of gene expression	Possible	(152)
11	20A	Global changes of gene expression	Possible	(153)
12	Emetine	Alterations of alternative gene splicing	Unknown	(161)

The list indicates whether an R-loop role has been established in molecular mechanisms reported for the G4 binders discussed in the text.

### Genome stability is challenged by perturbations of steady-state levels of G-loop structures

G-loops may occur in nuclear chromatin more often than previously recognized, and G4 binders may exert their biological activity through interference with these complex structures. An extensively-studied genomic site is the murine IgH locus where G-loops form during immunoglobulin class switch recombination (CSR) (94). R-loops and G4s form on the template and non-template strands, respectively, during transcription of switch regions to trigger DNA breaks and the recombination pathway. Interestingly, one function proposed for G-loops is related to the link between CSR and cell replication. Replication origins are present in switch sequences and G4s can function as a loading substrate for the recruitment of origin recognition complex (ORC) (119–121). R-loops have been shown to favor the physical proximity (synapsis) of replication origins firing at multiple sites within the 3–12 kb-long recombining switch regions. Therefore, R-loops may promote DSB resolution by regulating long-distance origin interactions, thus explaining the dependence of CSR on S phase and cell proliferation (122).

The mechanisms of transcription-induced genome instability has been investigated in yeast using the murine Sμ switch region (123,124), which can readily form G-loops during transcription. Using genetic screens, Topoisomerase I was identified as a critical factor in suppressing gross chromosomal rearrangements and loss of heterozygosity at the PQS of the switch region. It is known that Topoisomerase I can regulate R-loops likely by reducing negative supercoils of the template (51,72), however the authors documented that this enzyme function is not sufficient to fully suppress genome instability at PQS (124). Interestingly, Topoisomerase I candirectly bind to G4 structures *in vitro* (125,126), localize to telomeres in yeast (127), cleave telomeric G-rich repeats (128). In addition, the enzyme activity is inhibited by G4 aptamers (reviewed in (129)). Thus, the findings in yeast on the Sμ switch region may suggest a complex mechanism of G-loop regulation by topoisomerase I during transcription, which may involve the enzyme binding to G4 to recruit other factors, such as helicases, to resolve the structure and prevent gene rearrangements (124).

Unbalanced G4 stabilization is a consequence of mutation or cellular reduction of G4 resolvases, such as Pif1 and the RecQ-type Bloom (BLM) (4). Fibroblasts from BLM patients show significant genome instability in comparison to healthy fibroblasts, including a 10-fold increase in sister chromatid exchanges, which often occur at G4-promoting sequences and at fragile sites of transcribed genes (130). This may suggest that recombination at active genes might involve R-loops and is a major contributor to genome instability in BLM-deficient cells. Pif1, and related family members, are among the most active helicases in resolving G4 structures and have been shown in yeast to prevent replication-dependent genetic and epigenetic alterations (131,132). In these studies, the authors showed that stabilized G4s caused replication fork arrests in a much wider region than the PQS. As Pif1 is also very active in resolving DNA:RNA hybrids (4), it is therefore possible that G4 structures may contribute to the formation of longer G-loops, explaining the reported regional rather than site-specific fork arrests and the consequent impediment to the replication machinery (131).

Eukaryotic telomeric sequences can assemble into R-loops and G4s, suggesting that a functional interplay can occur between the two structures for telomere maintenance. Replication rate of telomeric DNAs is slower in the presence of PhenDC3, a G4 stabilizer (133), or BLM depletion (133). BLM-deficient cells exhibited more G4 structures in telomeres than BLM-proficient cells, and the data indicated that the RecQ helicases allowed telomere replication by modulating G4s during G-rich strand synthesis (133). Telomeric G4s may either compete or cooperate with R-loops depending on the mechanism. For instance, an RNA:DNA hybrid occurs at telomeres due to telomerase, a reverse transcriptase that uses an RNA molecule as substrate to extend the telomeric DNA. Then, telomeric repeat-containing RNA (TERRA) can form R-loops in trans at telomeres to trigger the repair of short telomeres via RAD51-mediated homology repair, as seen by DRIP-seq genome mapping (134–136). Interestingly, it has been demonstrated that ATRX (a G4-binding chromatin remodeling factor) can be recruited at tandem repeats with a high GC content, including telomeric repeats in a transcription dependent-manner (137). ATRX is recruited at telomeres to suppress replication stalling, DNA damage and recombination pathways. The report showed that the absence of ATRX causes an increase of co-transcriptional G4/R-loops at telomeres which can impair the proper maintenance of telomere structure (137). One can speculate that ATRX may be recruited at telomeres by telomeric G4s stabilized by the DNA:RNA hybrid on the opposite strand as in G-loops.

RTEL1 (regulator of telomere length) is a critical helicase for maintenance and regulation of telomere length (4). Its depletion impairs the disassembly of the telomere-protecting T-loop assembly (a lasso-like telomere organization) and enhances murine telomere fragility induced by the G4 binder TMPyP4 (138). Recent reports have extended the role of RTEL1 in protecting not only telomeres but the whole genome from replication/transcription conflicts at G-loop-forming regions (139,140). Interestingly, RETL1 helicase activity has a critical role in completing DNA duplication during mitosis at fragile loci prone to form G-loops. In the absence of RTEL1, cells accumulate G-loops at those sites, which remain under-replicated causing marked genome instability in daughter cells (140).

### G4/R-loop disturbance of replication can impair epigenetic memory

Recent findings connect DNA and RNA G4s to methylation of DNA and histone post-translational modifications, revealing novel functions of G4 structures in different mechanisms of chromatin and epigenetic regulation (reviewed in (3)). Interestingly, stabilization of G4s has been shown to impair epigenetic memory by gene silencing in a manner dependent on DNA replication. In the BU-1 locus of chicken DT40 cells, G4s can arrest processive replication at the leading strand causing an impairment of the coupling of DNA synthesis and histone recycling/nucleosome reassembly failing to propagate precisely the parental pattern of histone modifications (141,142). As several specialized DNA helicases (such as PIF1, WRN, and BLM) and TLS polymerases can replicate through G4 structures, it has been shown that the helicase FANCJ and the polymerase Rev1 and PrimPol are critical to prevent leading strand replication block by G4 structures at the chicken BU-1 locus (141,142). In particular, PrimPol can bind to G4s and is then able to reprime DNA synthesis at few bases downstream of the G4 structure. As PrimPol deletion causes epigenetic instability and loss of gene expression, G4s may often form impediments to leading strand replication (142). Interestingly, specific G4 binders can affect epigenetic memory at the chicken BU-1 locus leading to the proposal to use such compounds in epigenetic reprogramming therapies (143). G4 binders could increase replication inhibition specifically at the locus, triggering local epigenetic instability by causing a loss of H3K4me3 and subsequently an increase of DNA methylation (143). This emphasizes the possibility to exploit G4 binders as modulators of the epigenetic memory of specific somatic tissues in pathological contexts.

Epigenetic instability has also been reported to depend on R-loop levels at the same chicken BU-1 locus. In particular, increased genome-wide R-loop levels were detected upon PrimPol deletion and replication impediments caused by purine-rich repeat (GAA_10_) or PQS during S phase (144). RNaseH1 overexpression resulted in a marked reduction of epigenetic instability showing that R-loop formation enhanced the G4-dependent block of leading strand replication, while PrimPol repriming activity inhibited unscheduled R-loop formation (144). Although the structural relationships between the DNA:RNA hybrid and G4, or other secondary structures, needs to be fully clarified, it is possible that a G-loop may form during replication at the BU-1 locus of chicken DT40 cells. An elucidation of G4/R-loop structures at this locus in S-phase cells may also be relevant to better understand replication/transcription conflicts caused by oncogene-induced high transcription rates or by cell treatments with G4 binders and other antitumor drugs in cancer cells (60,145).

### G4 binders as triggers of altered gene expression programs

The stabilization of G4/R-loop structures by G4 interacting compounds may lead not only to replication arrest, DNA damage and genomic instability, but also to alterations of gene transcription. R-loops have been shown to pause RNA polymerases at different steps of the elongation process (62,64) and to regulate epigenetic mechanisms such as DNA methylation (60,146). As G4 binders can have a stabilizing effect on R-loops at active genes, this action may therefore explain transcription rate alterations observed with G4 binders. Interestingly, a recent report has investigated the structural and functional interplays of G4, R-loops and T7 RNA polymerase transcription using biophysical assays *in vitro* (147). The data show that transcription elongation efficiency depends on the relative orientation of PQS. In particular, when G4s form in the non-template strand, they increase the final RNA product level due to the formation of co-transcriptional R-loops. R-loop formation in turn favors the next round of T7 RNA polymerase binding to the promoter and, hence, transcription (147). On the other hand, G4 formation in the template strand can directly block RNA polymerases. As the mechanism of how G4 binders can modulate gene transcription is not yet fully defined in living cells, it is therefore of high interest to define R-loop roles in transcriptional effects of G4 binders in future studies.

Although G4s can likely form at a multitude of active gene promoters, fewer studies have determined the genome-wide effects of G4 stabilization. Depletion of BLM or WRN helicases, which likely leads to G4 stabilization, cause not only genome instability as discussed above but also specific alterations of gene expression profiles (148–150). This has mechanistically been ascribed to G4 regulation of transcription as reduced expression was observed mainly at genes harboring PQS. By studying the effects of a naphthalene diimide derivative, CM03 (Figure 2), using an RNA-seq approach on two pancreatic cancer cell lines, Marchetti et al. (151) showed after 6 h treatments a large down-regulation of many genes, which are rich in PQS and involved in essential pathways of pancreatic cancer cell survival and tumor progression. In another study (152), transcriptional expression profiles affected by the G4 binder AQ1 (Figure 2), an anthraquinone derivative developed to target the KIT promoter, were determined using an RNA-seq approach in canine and human cell lines. The findings confirmed the KIT gene expression down-regulation but also the down-regulation of MYC-related pathways and up-regulation of p53, apoptosis and hypoxia-response pathways in both species. Beauvarlet et al. (153) investigated the effects of the triarylpyridine derivative, 20A (Figure 2), on growth arrest of cancer cells and antitumor activity in mice. Gene expression profiles were altered by the ligand in a manner dependent on G4 density of genes. However, the ligand was able to induce global DNA damage activating an ATM-dependent response and autophagy pathways, which would affect gene expression as well. The authors showed that ATM depletion could markedly reduced autophagy and senescence leading cancer cells to death (153).

RNA G4s have been implicated in splicing regulation and several mechanisms of alternative splicing alterations due to intronic and/or exonic RNA G4 have been proposed at selected genes, such as hTERT, TP53 and FMR1 (154–156). G4 binders may also affect splicing regulation by interacting with G4s folded in the pre-mRNA during maturation. Studying emetine (Figure 2), recent bioinformatic analyses showed this G4 binder could have a global effect in regulating RNA G4-dependent alternative splicing. In particular, the authors found that 60% of emetine-regulated exon skipping events contained potential G4 structures proximal to the splice site (157).

Overall, these investigations demonstrate that genetic or chemical G4 stabilization can lead to reduced gene expression by inhibiting transcription or splicing, suggesting that G4 structures can constitute a barrier to RNA polymerase elongation. Steady-state levels of transcripts were often determined after long times of treatment, therefore the data can not distinguish the contribution of cell response mechanisms from the direct effects of the studied ligands on transcription elongation. Even though further investigations are needed to fully clarify the mechanisms, the findings overall show that G4 binders might exert a pharmacological activity by specifically altering gene expression programs of cancer cells.

Gene expression can also be altered by interfering with functions and/or stability of mRNAs and protein synthesis by ribosomes. Initially, studies on G4s focused on DNA, however these secondary structures can also form in RNA strands that are conformationally, more stable as the ribose 2′-hydroxyl groups establish new intramolecular interactions (158,159). The single-stranded nature of transcripts may likely favor G4 folding of G-rich sequences of RNAs *in vivo*. A critical role of RNA G4 in cellular process has been supported by computational analyses showing their enrichment at regulatory 5′- and 3′-UTRs, enzymatic and/or chemical foot-printing, largely used to reveal G4s in transcripts, and RNA G4 structure visualization in living cells (98,160–162). Many studies focusing at single gene transcripts, such as FMRP, KRAS, NRAS, BCL-2 and VEGF, provided data supporting ligand inhibition of translation by stabilizing G4 folding in the studied mRNA (163–167). High-throughput RNA sequencing technologies allowed RNA G4 mapping throughout the entire transcriptome (168), emphasizing a role in translation regulation (169), mRNA localization, turnover and metabolism (170,171). By using a specific G4-RNA chemical pull-down followed by a sequencing, it has been shown that transient G4 formation can occur in the human transcriptome and that two distinct G4 binders (Braco-19 and RHPS4, Figure 2) can influence the global G4 transcriptome landscape (172), documenting that they may exert a biological activity also by interfering with RNA G4-dependent regulation mechanisms.

Moreover, as for DNA G4s, RNA G4 helicases can play a role in mRNA regulation. By addressing genome-wide effects, the inhibition by a natural compound of the helicase activity of eukaryotic initiation factor 4A (eIF4A) resulted into the translational down-regulation of a subset of genes harboring PQS at their 5′-UTRs, which included oncogenes and super-enhancers-associated transcription factors (173). Other specific RNA helicases can also affect protein synthesis such as the cytoplasmic DHX36 helicase, which has been shown to promote mRNA translation (3).

Collectively, the findings show that DNA and RNA G4s can regulate mRNA structure, translation and overall gene expression, and G4 binders may thus alter gene expression programs of cancer cells. A common hallmark of cancer is the impairment of transcriptional regulation mechanisms that can generate a dependency of cancer cells to altered transcriptional processes (174). Moreover, cancer cells may be characterized by an overall enhanced level of gene expression, mainly due to a high transcription rate driven by c-myc oncogene amplification or overstimulation (175). In this context, on one hand, G4 and R-loop levels can be higher at cancer genes due to enhanced transcriptional activity (12,30). On the other, the cancer transcriptional addiction may be exploited to develop G4 binders more effective in down-regulating overall the cancer transcriptional program by targeting many G4 structures at gene as well as transcript levels.

## CONCLUSION

The double-stranded nature of the human genome is not permanent but changes dynamically to allow fundamental processes. This offers an opportunity to modulate cellular activity by small compounds able to bind specifically to non-canonical nucleic acid secondary structures, such as G4s or even R-loops. Evidence shows that G4s are not only structurally compatible with R-loops but they can form contextually in *in vitro* systems and living cells. R-loop levels can be increased by G4 binders in cancer cells and are needed for the induction of DNA damage, cell killing and genome instability. Even though non-R-loop-mediated pathways of DNA damage induction by G4s and/or G4 binders are known (Figure 4), R-loops may play a critical role in the mechanism of action of G4 binders and novel insights are likely to provide a new rationale to discover clinically-effective new anticancer G4 binders.

Several thousands G4s can likely form in DNA and RNA strands folding in several different conformations, however known G4 binders cannot bind to only one or few G4s, but rather they bind and stabilize different conformations and many genome-wide G4s. Thus, it is unlikely that known ligands can act at very few genomic loci. Several published data support a more general mechanism of action of known G4 binders that can induce DNA damage, genome and epigenome instabilities and modification of gene expression programs, thus exerting biological activities including cell death and proliferation arrest. Therefore, future efforts need to focus on specific targets, mechanisms of action and global ligand effects in the context of specific cell tissue types to get new insights for anticancer G4 binder discovery.

## References

[1] Mergny J.-L. , Sen D. DNA quadruple helices in nanotechnology. Chem. Rev. 2019; 119:6290–6325. 30605316 10.1021/acs.chemrev.8b00629

[2] Spiegel J. , Adhikari S., Balasubramanian S. The structure and function of DNA G-Quadruplexes. Trends Chem. 2020; 2:123–136. 32923997 10.1016/j.trechm.2019.07.002 PMC7472594

[3] Varshney D. , Spiegel J., Zyner K., Tannahill D., Balasubramanian S. The regulation and functions of DNA and RNA G-quadruplexes. Nat. Rev. Mol. Cell Biol. 2020; 21:459–474. 32313204 10.1038/s41580-020-0236-x PMC7115845

[4] Sauer M. , Paeschke K. G-quadruplex unwinding helicases and their function in vivo. Biochem. Soc. Trans. 2017; 45:1173–1182. 28939694 10.1042/BST20170097

[5] Capra J.A. , Paeschke K., Singh M., Zakian V.A. G-Quadruplex DNA sequences are evolutionarily conserved and associated with distinct genomic features in Saccharomyces cerevisiae. PLoS Comput. Biol. 2010; 6:e1000861. 20676380 10.1371/journal.pcbi.1000861 PMC2908698

[6] Puig Lombardi E. , Holmes A., Verga D., Teulade-Fichou M.-P., Nicolas A., Londoño-Vallejo A. Thermodynamically stable and genetically unstable G-quadruplexes are depleted in genomes across species. Nucleic Acids Res. 2019; 47:6098–6113. 31114920 10.1093/nar/gkz463 PMC6614823

[7] Ruggiero E. , Richter S.N. G-quadruplexes and G-quadruplex ligands: targets and tools in antiviral therapy. Nucleic Acids Res. 2018; 46:3270–3283. 29554280 10.1093/nar/gky187 PMC5909458

[8] Salgado G.F. , Cazenave C., Kerkour A., Mergny J.-L. G-quadruplex DNA and ligand interaction in living cells using NMR spectroscopy. Chem. Sci. 2015; 6:3314–3320. 28706695 10.1039/c4sc03853c PMC5490339

[9] Kwok C.K. , Merrick C.J., Kwok C.K., Uk C.M.A., Merrick C.J. G-Quadruplexes: prediction, characterization, and biological application. Trends Biotechnol. 2017; 35:997–1013. 28755976 10.1016/j.tibtech.2017.06.012

[10] Carvalho J. , Mergny J.-L., Salgado G.F., Queiroz J.A., Cruz C. G-quadruplex, friend or foe: the role of the G-quartet in anticancer strategies. Trends Mol. Med. 2020; 26:848–861. 32467069 10.1016/j.molmed.2020.05.002

[11] Balasubramanian S. , Hurley L.H., Neidle S. Targeting G-quadruplexes in gene promoters: a novel anticancer strategy. Nat. Rev. Drug Discov. 2011; 10:261–275. 21455236 10.1038/nrd3428 PMC3119469

[12] Hänsel-Hertsch R. , Di Antonio M., Balasubramanian S. DNA G-quadruplexes in the human genome: detection, functions and therapeutic potential. Nat. Rev. Mol. Cell Biol. 2017; 18:279–284. 28225080 10.1038/nrm.2017.3

[13] Wu G. , Chen L., Liu W., Yang D. Molecular recognition of the hybrid-type G-quadruplexes in human telomeres. Molecules. 2019; 24:1578. 10.3390/molecules24081578 PMC6514847 31013622

[14] Zuffo M. , Guédin A., Leriche E.-D., Doria F., Pirota V., Gabelica V., Mergny J.-L., Freccero M. More is not always better: finding the right trade-off between affinity and selectivity of a G-quadruplex ligand. Nucleic Acids Res. 2018; 46:e115. 29986058 10.1093/nar/gky607 PMC6212845

[15] Asamitsu S. , Obata S., Yu Z., Bando T., Sugiyama H. Recent progress of targeted G-Quadruplex-Preferred ligands toward cancer therapy. Molecules. 2019; 24:429. 10.3390/molecules24030429 PMC6384606 30682877

[16] Neidle S. Quadruplex nucleic acids as novel therapeutic targets. J. Med. Chem. 2016; 59:5987–6011. 26840940 10.1021/acs.jmedchem.5b01835

[17] Pagano A. , Iaccarino N., Abdelhamid M.A.S., Brancaccio D., Garzarella E.U., Di Porzio A., Novellino E., Waller Z.A.E., Pagano B., Amato J. et al. Common G-Quadruplex binding agents found to interact with i-motif-forming DNA: unexpected multi-target-directed compounds. Front. Chem. 2018; 6:281. 30137743 10.3389/fchem.2018.00281 PMC6066642

[18] Bruno P.M. , Lu M., Dennis K.A., Inam H., Moore C.J., Sheehe J., Elledge S.J., Hemann M.T., Pritchard J.R. The primary mechanism of cytotoxicity of the chemotherapeutic agent CX-5461 is topoisomerase II poisoning. Proc. Natl. Acad. Sci. USA. 2020; 117:4053–4060. 32041867 10.1073/pnas.1921649117 PMC7049172

[19] Olivieri M. , Cho T., Álvarez-Quilón A., Li K., Schellenberg M.J., Zimmermann M., Hustedt N., Rossi S.E., Adam S., Melo H. et al. A genetic map of the response to DNA damage in human cells. Cell. 2020; 182:481–496. 32649862 10.1016/j.cell.2020.05.040 PMC7384976

[20] Lerner L.K. , Sale J.E. Replication of G quadruplex DNA. Genes (Basel). 2019; 10:95. 10.3390/genes10020095 PMC6409989 30700033

[21] Bryan T.M. Mechanisms of DNA replication and repair: insights from the study of G-quadruplexes. Molecules. 2019; 24:3439. 10.3390/molecules24193439 PMC6804030 31546714

[22] Maizels N. G4-associated human diseases. EMBO Rep. 2015; 16:910–922. 26150098 10.15252/embr.201540607 PMC4552485

[23] Puget N. , Miller K.M., Legube G. Non-canonical DNA/RNA structures during transcription-coupled double-strand break repair: roadblocks or bona fide repair intermediates. DNA Repair (Amst.). 2019; 81:102661. 31331819 10.1016/j.dnarep.2019.102661 PMC6764918

[24] Huppert J.L. , Balasubramanian S. G-quadruplexes in promoters throughout the human genome. Nucleic Acids Res. 2007; 35:406–413. 17169996 10.1093/nar/gkl1057 PMC1802602

[25] Huppert J.L. , Balasubramanian S. Prevalence of quadruplexes in the human genome. Nucleic Acids Res. 2005; 33:2908–2916. 15914667 10.1093/nar/gki609 PMC1140081

[26] Todd A.K. , Johnston M., Neidle S. Highly prevalent putative quadruplex sequence motifs in human DNA. Nucleic Acids Res. 2005; 33:2901–2907. 15914666 10.1093/nar/gki553 PMC1140077

[27] Chambers V.S. , Marsico G., Boutell J.M., Di Antonio M., Smith G.P., Balasubramanian S. High-throughput sequencing of DNA G-quadruplex structures in the human genome. Nat. Biotechnol. 2015; 33:877–881. 26192317 10.1038/nbt.3295

[28] Marsico G. , Chambers V.S., Sahakyan A.B., McCauley P., Boutell J.M., Di Antonio M., Balasubramanian S. Whole genome experimental maps of DNA G-quadruplexes in multiple species. Nucleic Acids Res. 2019; 47:3862–3874. 30892612 10.1093/nar/gkz179 PMC6486626

[29] Hänsel-Hertsch R. , Beraldi D., Lensing S.V, Marsico G., Zyner K., Parry A., Di Antonio M., Pike J., Kimura H., Narita M. et al. G-quadruplex structures mark human regulatory chromatin. Nat. Genet. 2016; 48:1267–1272. 27618450 10.1038/ng.3662

[30] Kouzine F. , Wojtowicz D., Baranello L., Yamane A., Nelson S., Resch W., Kieffer-Kwon K.R., Benham C.J., Casellas R., Przytycka T.M. et al. Permanganate/S1 nuclease footprinting reveals Non-B DNA structures with regulatory potential across a mammalian genome. Cell Syst. 2017; 4:344–356. 28237796 10.1016/j.cels.2017.01.013 PMC7432990

[31] Risitano A. , Fox K.R. Stability of intramolecular DNA quadruplexes: comparison with DNA duplexes ^†^. Biochemistry. 2003; 42:6507–6513. 12767234 10.1021/bi026997v

[32] Mendoza O. , Elezgaray J., Mergny J.-L. Kinetics of quadruplex to duplex conversion. Biochimie. 2015; 118:225–233. 26427557 10.1016/j.biochi.2015.09.031

[33] Zhou J. , Wei C., Jia G., Wang X., Feng Z., Li C. Human telomeric G-quadruplex formed from duplex under near physiological conditions: Spectroscopic evidence and kinetics. Biochimie. 2009; 91:1104–1111. 19524012 10.1016/j.biochi.2009.05.014

[34] Shirude P.S. , Okumus B., Ying L., Ha T., Balasubramanian S. Single-molecule conformational analysis of G-Quadruplex formation in the promoter DNA duplex of the Proto-Oncogene C-Kit. JACS. 2007; 129:7484–7485. 10.1021/ja070497d PMC2195893 17523641

[35] Liu L.F. , Wang J.C. Supercoiling of the DNA template during transcription. Proc. Nati. Acad. Sci. U.S.A. 1987; 84:7024–7027. 10.1073/pnas.84.20.7024 PMC299221 2823250

[36] Sekibo D.A.T. , Fox K.R. The effects of DNA supercoiling on G-quadruplex formation. Nucleic Acids Res. 2017; 45:12069–12079. 29036619 10.1093/nar/gkx856 PMC5716088

[37] Kouzine F. , Gupta A., Baranello L., Wojtowicz D., Ben-Aissa K., Liu J., Przytycka T.M., Levens D. Transcription-dependent dynamic supercoiling is a short-range genomic force. Nat. Struct. Mol. Biol. 2013; 20:396–403. 23416947 10.1038/nsmb.2517 PMC3594045

[38] Azorin F. , Nordheim A., Rich A. Formation of Z-DNA in negatively supercoiled plasmids is sensitive to small changes in salt concentration within the physiological range. EMBO J. 1983; 2:649–655. 6315414 10.1002/j.1460-2075.1983.tb01479.x PMC555164

[39] Mirkin S. , Frank-Kamenetskii M. H-DNA and related structures. Annu. Rev. Biophys. Biomol. Struct. 1994; 23:541–576. 7919793 10.1146/annurev.bb.23.060194.002545

[40] Frelih T. , Wang B., Plavec J., Šket P. Pre-folded structures govern folding pathways of human telomeric G-quadruplexes. Nucleic Acids Res. 2020; 48:2189–2197. 31950178 10.1093/nar/gkz1235 PMC7038944

[41] Sun D. , Hurley L.H. The importance of negative superhelicity in inducing the formation of G-quadruplex and i-motif structures in the c-Myc promoter: implications for drug targeting and control of gene expression. J. Med. Chem. 2009; 52:2863–2874. 19385599 10.1021/jm900055s PMC2757002

[42] Onyshchenko M.I. , Gaynutdinov T.I., Englund E.A., Appella D.H., Neumann R.D., Panyutin I.G. Quadruplex formation is necessary for stable PNA invasion into duplex DNA of BCL2 promoter region. Nucleic Acids Res. 2011; 39:7114–7123. 21593130 10.1093/nar/gkr259 PMC3167611

[43] Dhakal S. , Yu Z., Konik R., Cui Y., Koirala D., Mao H. G-quadruplex and i-motif are mutually exclusive in ILPR double-stranded DNA. Biophys. J. 2012; 102:2575–2584. 22713573 10.1016/j.bpj.2012.04.024 PMC3368138

[44] Cui Y. , Koirala D., Kang H., Dhakal S., Yangyuoru P., Hurley L.H., Mao H. Molecular population dynamics of DNA structures in a bcl-2 promoter sequence is regulated by small molecules and the transcription factor hnRNP LL. Nucleic Acids Res. 2014; 42:5755–5764. 24609386 10.1093/nar/gku185 PMC4027204

[45] Kang H.-J. , Kendrick S., Hecht S.M., Hurley L.H. The transcriptional complex between the BCL2 i-Motif and hnRNP LL is a molecular switch for control of gene expression that can be modulated by small molecules. J. Am. Chem. Soc. 2014; 136:4172–4185. 24559432 10.1021/ja4109352 PMC3985447

[46] Levens D. , Baranello L., Kouzine F. Controlling gene expression by DNA Mechanics: emerging insights and challenges. Biophys. Rev. 2016; 8:259–268. 28510225 10.1007/s12551-016-0216-8 PMC5425794

[47] Zhabinskaya D. , Benham C.J. Competitive superhelical transitions involving cruciform extrusion. Nucleic Acids Res. 2013; 41:9610–9621. 23969416 10.1093/nar/gkt733 PMC3834812

[48] Liu J. , Kouzine F., Nie Z., Chung H.J., Elisha-Feil Z., Weber A., Zhao K., Levens D. The FUSE/FBP/FIR/TFIIH system is a molecular machine programming a pulse of c-myc expression. EMBO J. 2006; 25:2119–2130. 16628215 10.1038/sj.emboj.7601101 PMC1462968

[49] Huppert J.L. Thermodynamic prediction of RNA–DNA duplex-forming regions in the human genome. Mol. Biosyst. 2008; 4:686–691. 18493667 10.1039/b800354h

[50] Roberts R. , Crothers D. Stability and properties of double and triple Helices: dramatic effects of RNA or DNA backbone composition. Science (80-.). 1992; 258:1463–1469. 10.1126/science.1279808 1279808

[51] Manzo S.G. , Hartono S.R., Sanz L.A., Marinello J., De Biasi S., Cossarizza A., Capranico G., Chedin F. DNA topoisomerase I differentially modulates R-loops across the human genome. Genome Biol. 2018; 19:100. 30060749 10.1186/s13059-018-1478-1 PMC6066927

[52] Chedin F. Nascent connections: R-loops and chromatin patterning. Trends Genet. 2016; 32:828–838. 27793359 10.1016/j.tig.2016.10.002 PMC5123964

[53] Richardson J.P. Attachment of nascent RNA molecules to SuperheHcal DNA. J. Mol. Biol. 1975; 98:566–579. 10.1016/s0022-2836(75)80087-8 811809

[54] Drolet M. , Bi X., Liu L.F. Hypernegative supercoiling of the DNA template during transcription elongation in vitro. J. Biol. Chem. 1994; 269:2068–2074. 8294458

[55] Drolet M. , Phoenix P., Menzel R., Masse E., Liu L.F., Crouch R.J. Overexpression of RNase H partially complements the growth defect of an Escherichia coli delta topA mutant: R-loop formation is a major problem in the absence of DNA topoisomerase I. Proc. Natl. Acad. Sci. U.S.A. 1995; 92:3526–3530. 7536935 10.1073/pnas.92.8.3526 PMC42200

[56] Drolet M. Growth inhibition mediated by excess negative supercoiling: the interplay between transcription elongation, R-loop formation and DNA topology. Mol. Microbiol. 2006; 59:723–730. 16420346 10.1111/j.1365-2958.2005.05006.x

[57] Usongo V. , Martel M., Balleydier A.A., Drolet M. Mutations reducing replication from R-loops suppress the defects of growth, chromosome segregation and DNA supercoiling in cells lacking topoisomerase I and RNase HI activity. DNA Repair (Amst.). 2016; 40:1–17. 26947024 10.1016/j.dnarep.2016.02.001

[58] Masse E. , Drolet M. Escherichia coli DNA topoisomerase I inhibits R-loop formation by relaxing transcription-induced negative supercoiling. J. Biol. Chem. 1999; 274:16659–16664. 10347234 10.1074/jbc.274.23.16659

[59] Masse E. , Phoenix P., Drolet M. DNA topoisomerases regulate R-loop formation during transcription of the rrnB operon in Escherichia coli. J. Biol. Chem. 1997; 272:12816–12823. 9139742 10.1074/jbc.272.19.12816

[60] Santos-Pereira J.M. , Aguilera A. R loops: new modulators of genome dynamics and function. Nat. Rev. Genet. 2015; 16:583–597. 26370899 10.1038/nrg3961

[61] Crossley M.P. , Bocek M., Cimprich K.A. R-Loops as cellular regulators and genomic threats. Mol. Cell. 2019; 73:398–411. 30735654 10.1016/j.molcel.2019.01.024 PMC6402819

[62] Skourti-Stathaki K. , Proudfoot N.J. A double-edged sword: R loops as threats to genome integrity and powerful regulators of gene expression. Genes Dev. 2014; 28:1384–1396. 24990962 10.1101/gad.242990.114 PMC4083084

[63] Naughton C. , Avlonitis N., Corless S., Prendergast J.G., Mati I.K., Eijk P.P., Cockroft S.L., Bradley M., Ylstra B., Gilbert N. Transcription forms and remodels supercoiling domains unfolding large-scale chromatin structures. Nat. Struct. Mol. Biol. 2013; 20:387–395. 23416946 10.1038/nsmb.2509 PMC3689368

[64] Chedin F. , Benham C.J. Emerging roles for R-loop structures in the management of topological stress. J. Biol. Chem. 2020; 295:4684–4695. 32107311 10.1074/jbc.REV119.006364 PMC7135976

[65] Wahba L. , Costantino L., Tan F.J., Zimmer A., Koshland D. S1-DRIP-seq identifies high expression and polyA tracts as major contributors to R-loop formation. Genes Dev. 2016; 30:1327–1338. 27298336 10.1101/gad.280834.116 PMC4911931

[66] Ginno P.A.A. , Lott P.L.L., Christensen H.C.C., Korf I., Chédin F. R-loop formation is a distinctive characteristic of unmethylated human CpG Island promoters. Mol. Cell. 2012; 45:814–825. 22387027 10.1016/j.molcel.2012.01.017 PMC3319272

[67] Sanz L.A. , Hartono S.R., Lim Y.W., Steyaert S., Rajpurkar A., Ginno P.A., Xu X., Chedin F. Prevalent, dynamic, and conserved R-Loop structures associate with specific epigenomic signatures in mammals. Mol. Cell. 2016; 63:167–178. 27373332 10.1016/j.molcel.2016.05.032 PMC4955522

[68] Stork C.T. , Bocek M., Crossley M.P., Sollier J., Sanz L.A., Chedin F., Swigut T., Cimprich K.A. Co-transcriptional R-loops are the main cause of estrogen-induced DNA damage. Elife. 2016; 5:e17548. 27552054 10.7554/eLife.17548 PMC5030092

[69] Xu W. , Xu H., Li K., Fan Y., Liu Y., Yang X., Sun Q. The R-loop is a common chromatin feature of the Arabidopsis genome. Nat. Plants. 2017; 3:704–714. 28848233 10.1038/s41477-017-0004-x

[70] El Hage A. , Webb S., Kerr A., Tollervey D. Genome-wide distribution of RNA-DNA hybrids identifies RNase H targets in tRNA genes, retrotransposons and mitochondria. PLos Genet. 2014; 10:e1004716. 25357144 10.1371/journal.pgen.1004716 PMC4214602

[71] De Magis A. , Manzo S.G., Russo M., Marinello J., Morigi R., Sordet O., Capranico G. DNA damage and genome instability by G-quadruplex ligands are mediated by R loops in human cancer cells. Proc. Natl. Acad. Sci. USA. 2019; 116:816–825. 30591567 10.1073/pnas.1810409116 PMC6338839

[72] El Hage A. , French S.L., Beyer A.L., Tollervey D. Loss of topoisomerase I leads to R-loop-mediated transcriptional blocks during ribosomal RNA synthesis. Genes Dev. 2010; 24:1546–1558. 20634320 10.1101/gad.573310 PMC2904944

[73] Shafiq S. , Chen C., Yang J., Cheng L., Ma F., Widemann E., Sun Q. DNA topoisomerase 1 prevents R-loop accumulation to modulate auxin-regulated root development in rice. Mol. Plant. 2017; 10:821–833. 28412545 10.1016/j.molp.2017.04.001

[74] Baranello L. , Wojtowicz D., Cui K., Devaiah B.N.N., Chung H.-J., Chan-Salis K.Y., Guha R., Wilson K., Zhang X., Zhang H. et al. RNA polymerase II regulates topoisomerase 1 activity to favor efficient transcription. Cell. 2016; 165:357–371. 27058666 10.1016/j.cell.2016.02.036 PMC4826470

[75] Duquette M.L. , Handa P., Vincent J.A., Taylor A.F., Maizels N. Intracellular transcription of G-rich DNAs induces formation of G-loops, novel structures containing G4 DNA. Genes Dev. 2004; 18:1618–1629. 15231739 10.1101/gad.1200804 PMC443523

[76] Zhao Y. , Zhang J.-Y., Zhang Z.-Y., Tong T.-J., Hao Y.-H., Tan Z. Real-Time detection reveals responsive cotranscriptional formation of persistent intramolecular DNA and intermolecular DNA:RNA hybrid G‐quadruplexes stabilized by R‐Loop. Anal. Chem. 2017; 89:6036–6042. 28447783 10.1021/acs.analchem.7b00625

[77] Neaves K.J. , Huppert J.L., Henderson R.M., Edwardson J.M. Direct visualization of G-quadruplexes in DNA using atomic force microscopy. Nucleic Acids Res. 2009; 37:6269–6275. 19696072 10.1093/nar/gkp679 PMC2764456

[78] Carrasco-Salas Y. , Malapert A., Sulthana S., Molcrette B., Chazot-Franguiadakis L., Bernard P., Chédin F., Faivre-Moskalenko C., Vanoosthuyse V. The extruded non-template strand determines the architecture of R-loops. Nucleic Acids Res. 2019; 47:6783–6795. 31066439 10.1093/nar/gkz341 PMC6648340

[79] Zhang M. , Wang B., Li T., Liu R., Xiao Y., Geng X., Li G., Liu Q., Price C.M., Liu Y. et al. Mammalian CST averts replication failure by preventing G-quadruplex accumulation. Nucleic Acids Res. 2019; 47:5243–5259. 30976812 10.1093/nar/gkz264 PMC6547417

[80] Zeman M.K. , Cimprich K.A. Causes and consequences of replication stress. Nat. Cell Biol. 2014; 16:2–9. 24366029 10.1038/ncb2897 PMC4354890

[81] Maréchal A. , Zou L. RPA-coated single-stranded DNA as a platform for post-translational modifications in the DNA damage response. Cell Res. 2015; 25:9–23. 25403473 10.1038/cr.2014.147 PMC4650586

[82] Nguyen H.D. , Yadav T., Giri S., Saez B., Graubert T.A., Zou L. Functions of replication protein A as a sensor of R Loops and a regulator of RNaseH1. Mol. Cell. 2017; 65:832–847. 28257700 10.1016/j.molcel.2017.01.029 PMC5507214

[83] Sun Q. , Csorba T., Skourti-Stathaki K., Proudfoot N.J., Dean C. R-Loop stabilization represses antisense transcription at the arabidopsis FLC locus. Science (80-.). 2013; 340:619–621. 10.1126/science.1234848 PMC5144995 23641115

[84] Mendoza O. , Bourdoncle A., Boulé J.B., Brosh R.M., Mergny J.L. G-quadruplexes and helicases. Nucleic Acids Res. 2016; 44:1989–2006. 26883636 10.1093/nar/gkw079 PMC4797304

[85] Wanrooij P.H. , Uhler J.P., Shi Y., Westerlund F., Falkenberg M., Gustafsson C.M. A hybrid G-quadruplex structure formed between RNA and DNA explains the extraordinary stability of the mitochondrial R-loop. Nucleic Acids Res. 2012; 40:10334–10344. 22965135 10.1093/nar/gks802 PMC3488243

[86] Zheng K.-W. , Wu R.-Y., He Y.-D., Xiao S., Zhang J.-Y., Liu J.-Q., Hao Y.-H., Tan Z. A competitive formation of DNA:RNA hybrid G-quadruplex is responsible to the mitochondrial transcription termination at the DNA replication priming site. Nucleic Acids Res. 2014; 42:10832–10844. 25140009 10.1093/nar/gku764 PMC4176368

[87] Falabella M. , Kolesar J.E., Wallace C., de Jesus D., Sun L., Taguchi Y.V., Wang C., Wang T., Xiang I.M., Alder J.K. et al. G-quadruplex dynamics contribute to regulation of mitochondrial gene expression. Sci. Rep. 2019; 9:5605. 30944353 10.1038/s41598-019-41464-y PMC6447596

[88] Zheng K. , Xiao S., Liu J., Zhang J., Hao Y., Tan Z. Co-transcriptional formation of DNA:RNA hybrid G-quadruplex and potential function as constitutional cis element for transcription control. Nucleic Acids Res. 2013; 41:5533–5541. 23585281 10.1093/nar/gkt264 PMC3664831

[89] Zhang J. , Zheng K., Xiao S., Hao Y., Tan Z. Mechanism and manipulation of DNA:RNA hybrid G-quadruplex formation in transcription of G-Rich DNA. J. Am. Chem. Soc. 2014; 136:1381–1390. 24392825 10.1021/ja4085572

[90] Xu Y. , Ishizuka T., Yang J., Ito K., Katada H., Komiyama M., Hayashi T. Oligonucleotide models of telomeric DNA and RNA form a Hybrid G-quadruplex structure as a potential component of telomeres. J. Biol. Chem. 2012; 287:41787–41796. 23012368 10.1074/jbc.M112.342030 PMC3516727

[91] Bao H.-L. , Xu Y. Telomeric DNA-RNA-hybrid G-quadruplex exists in environmental conditions of HeLa cells. Chem. Commun. 2020; 56:6547. 10.1039/d0cc02053b 32396161

[92] Ribeiro de Almeida C. , Dhir S., Dhir A., Moghaddam A.E., Sattentau Q., Meinhart A., Proudfoot N.J. RNA helicase DDX1 converts RNA G-quadruplex structures into R-Loops to promote IgH class switch recombination. Mol. Cell. 2018; 70:650–662. 29731414 10.1016/j.molcel.2018.04.001 PMC5971202

[93] Qiao Q. , Wang L., Meng F.-L., Hwang J.K., Alt F.W., Correspondence H.W. AID recognizes structured DNA for class switch recombination. Mol. Cell. 2017; 67:361–373. 28757211 10.1016/j.molcel.2017.06.034 PMC5771415

[94] Maizels N. Immunoglobulin gene diversification. Annu. Rev. Genet. 2005; 39:23–46. 16285851 10.1146/annurev.genet.39.073003.110544

[95] Pucella J.N. , Chaudhuri J. AID invited to the G4 summit. Mol. Cell. 2017; 67:355–357. 28777947 10.1016/j.molcel.2017.07.020

[96] Dalloul Z. , Chenuet P., Dalloul I., Boyer F., Aldigier J.-C., Laffleur B., El Makhour Y., Ryffel B., Quesniaux V.F.J., Togbé D. et al. G-quadruplex DNA targeting alters class-switch recombination in B cells and attenuates allergic inflammation. J. Allergy Clin. Immunol. 2018; 142:1352–1355. 29935221 10.1016/j.jaci.2018.06.011

[97] Biffi G. , Tannahill D., McCafferty J., Balasubramanian S. Quantitative visualization of DNA G-quadruplex structures in human cells. Nat. Chem. 2013; 5:182–186. 23422559 10.1038/nchem.1548 PMC3622242

[98] Biffi G. , Di Antonio M., Tannahill D., Balasubramanian S. Visualization and selective chemical targeting of RNA G-quadruplex structures in the cytoplasm of human cells. Nat. Chem. 2014; 6:75–80. 24345950 10.1038/nchem.1805 PMC4081541

[99] Boguslawski S.J. , Smith D.E., Michalak M.A., Mickelson K.E., Yehle C.O., Patterson W.L., Carrico R.J. Characterization of monoclonal antibody to DNA.RNA and its application to immunodetection of hybrids. J. Immunol. Methods. 1986; 89:123–130. 2422282 10.1016/0022-1759(86)90040-2

[100] Phillips D.D. , Garboczi D.N., Singh K., Hu Z., Leppla S.H., Leysath C.E. The sub-nanomolar binding of DNA-RNA hybrids by the single chain Fv fragment of antibody S9.6. J. Mol. Recognit. 2013; 26:2166–2171. 10.1002/jmr.2284 PMC4061737 23784994

[101] Amato J. , Miglietta G., Morigi R., Iaccarino N., Locatelli A., Leoni A., Novellino E., Pagano B., Capranico G., Randazzo A. Monohydrazone based G-Quadruplex selective ligands induce DNA damage and genome instability in human cancer cells. J. Med. Chem. 2020; 63:3090–3103. 32142285 10.1021/acs.jmedchem.9b01866 PMC7997572

[102] Marinello J. , Chillemi G., Bueno S., Manzo S.G., Capranico G. Antisense transcripts enhanced by camptothecin at divergent CpG-island promoters associated with bursts of topoisomerase I-DNA cleavage complex and R-loop formation. Nucleic Acids Res. 2013; 41:10110–10123. 23999093 10.1093/nar/gkt778 PMC3905886

[103] Marinello J. , Bertoncini S., Aloisi I., Cristini A., Malagoli Tagliazucchi G., Forcato M., Sordet O., Capranico G. Dynamic effects of topoisomerase I inhibition on R-Loops and short transcripts at active promoters. PLoS One. 2016; 11:e0147053. 26784695 10.1371/journal.pone.0147053 PMC4718701

[104] Capranico G. , Marinello J., Baranello L. Dissecting the transcriptional functions of human DNA topoisomerase I by selective inhibitors: Implications for physiological and therapeutic modulation of enzyme activity. Biochim. Biophys. Acta - Rev. Cancer. 2010; 1806:240–250. 10.1016/j.bbcan.2010.06.003 20600630

[105] Maffia A. , Ranise C., Sabbioneda S. From R-loops to G-quadruplexes: emerging new threats for the replication Fork. Int. J. Mol. Sci. 2020; 21:1506. 10.3390/ijms21041506 PMC7073102 32098397

[106] Masuda-Sasa T. , Polaczek P., Peng X.P., Chen L., Campbell J.L. Processing of G4 DNA by Dna2 helicase/nuclease and replication protein A (RPA) provides insights into the mechanism of Dna2/RPA substrate recognition. J. Biol. Chem. 2008; 283:24359–24373. 18593712 10.1074/jbc.M802244200 PMC2528986

[107] Lin W. , Sampathi S., Dai H., Liu C., Zhou M., Hu J., Huang Q., Campbell J., Shin-Ya K., Zheng L. et al. Mammalian DNA2 helicase/nuclease cleaves G-quadruplex DNA and is required for telomere integrity. EMBO J. 2013; 32:1425–1439. 23604072 10.1038/emboj.2013.88 PMC3655473

[108] Salvati E. , Leonetti C., Rizzo A., Scarsella M., Mottolese M., Galati R., Sperduti I., Stevens M.F.G., D’Incalci M., Blasco M. et al. Telomere damage induced by the G-quadruplex ligand RHPS4 has an antitumor effect. J. Clin. Invest. 2007; 117:3236–3247. 17932567 10.1172/JCI32461 PMC2000812

[109] Rizzo A. , Salvati E., Porru M., D’Angelo C., Stevens M.F., D’Incalci M., Leonetti C., Gilson E., Zupi G., Biroccio A. Stabilization of quadruplex DNA perturbs telomere replication leading to the activation of an ATR-dependent ATM signaling pathway. Nucleic Acids Res. 2009; 37:5353–5364. 19596811 10.1093/nar/gkp582 PMC2760797

[110] Gauthier L.R. , Granotier C., Hoffschir F., Etienne O., Ayouaz A., Desmaze C., Mailliet P., Biard D.S., Boussin F.D. Rad51 and DNA-PKcs are involved in the generation of specific telomere aberrations induced by the quadruplex ligand 360A that impair mitotic cell progression and lead to cell death. Cell. Mol. Life Sci. 2012; 69:629–640. 21773671 10.1007/s00018-011-0767-6 PMC3265728

[111] Rodriguez R. , Miller K.M., Forment J.V, Bradshaw C.R., Nikan M., Britton S., Oelschlaegel T., Xhemalce B., Balasubramanian S., Jackson S.P. Small-molecule-induced DNA damage identifies alternative DNA structures in human genes. Nat. Chem. Biol. 2012; 8:301–310. 22306580 10.1038/nchembio.780 PMC3433707

[112] Zimmer J. , Tacconi E.M.C.C., Folio C., Badie S., Porru M., Klare K., Tumiati M., Markkanen E., Halder S., Ryan A. et al. Targeting BRCA1 and BRCA2 Deficiencies with G-Quadruplex-Interacting Compounds. Mol. Cell. 2016; 61:449–460. 26748828 10.1016/j.molcel.2015.12.004 PMC4747901

[113] Lemmens B. , van Schendel R., Tijsterman M. Mutagenic consequences of a single G-quadruplex demonstrate mitotic inheritance of DNA replication fork barriers. Nat. Commun. 2015; 6:8909. 26563448 10.1038/ncomms9909 PMC4654259

[114] van Bostelen I. , van Schendel R., Romeijn R., Tijsterman M. Translesion synthesis polymerases are dispensable for C. elegans reproduction but suppress genome scarring by polymerase theta-mediated end joining. PLos Genet. 2020; 16:e1008759. 32330130 10.1371/journal.pgen.1008759 PMC7202663

[115] Tian M. , Alt F.W. Transcription-induced cleavage of immunoglobulin switch regions by nucleotide excision repair nucleases in vitro. J. Biol. Chem. 2000; 275:24163–24172. 10811812 10.1074/jbc.M003343200

[116] Sollier J. , Stork C.T., García-Rubio M.L., Paulsen R.D., Aguilera A., Cimprich K.A. Transcription-coupled nucleotide excision repair factors promote R-loop-Induced genome instability. Mol. Cell. 2014; 56:777–785. 25435140 10.1016/j.molcel.2014.10.020 PMC4272638

[117] Cristini A. , Ricci G., Britton S., Salimbeni S., Huang S., Marinello J., Calsou P., Pommier Y., Favre G., Capranico G. et al. Dual processing of R-Loops and topoisomerase I induces transcription-dependent DNA double-strand breaks. Cell Rep. 2019; 28:3167–3181. 31533039 10.1016/j.celrep.2019.08.041 PMC8274950

[118] Yasuhara T. , Kato R., Hagiwara Y., Nakada S., Shibata A., Miyagawa K. Human Rad52 promotes XPG-Mediated R-loop processing to initiate transcription-associated homologous recombination repair. Cell. 2018; 175:558–570. 30245011 10.1016/j.cell.2018.08.056

[119] Hoshina S. , Yura K., Teranishi H., Kiyasu N., Tominaga A., Kadoma H., Nakatsuka A., Kunichika T., Obuse C., Waga S. Human origin recognition complex binds preferentially to G-quadruplex-preferable RNA and single-stranded DNA. J. Biol. Chem. 2013; 288:30161–30171. 24003239 10.1074/jbc.M113.492504 PMC3798484

[120] Valton A.L. , Hassan-Zadeh V., Lema I., Boggetto N., Alberti P., Saintomé C., Riou J.-F., Prioleau M.-N. G4 motifs affect origin positioning and efficiency in two vertebrate replicators. EMBO J. 2014; 33:732–746. 24521668 10.1002/embj.201387506 PMC4000090

[121] Lombraña R. , Almeida R., Álvarez A., Gómez M. R-loops and initiation of DNA replication in human cells: a missing link. Front. Genet. 2015; 6:158. 25972891 10.3389/fgene.2015.00158 PMC4412123

[122] Wiedemann E.-M. , Peycheva M., Pavri R. DNA replication origins in immunoglobulin switch regions regulate class switch recombination in an R-Loop-Dependent manner. Cell Rep. 2016; 17:2927–2942. 27974207 10.1016/j.celrep.2016.11.041

[123] Yadav P. , Harcy V., Argueso J.L., Dominska M., Jinks-robertson S., Kim N. Topoisomerase I plays a critical role in suppressing genome instability at a highly transcribed G-quadruplex-Forming sequence. PLos Genet. 2014; 10:e1004839. 25473964 10.1371/journal.pgen.1004839 PMC4256205

[124] Yadav P. , Owiti N., Kim N. The role of topoisomerase I in suppressing genome instability associated with a highly transcribed guanine-rich sequence is not restricted to preventing RNA:DNA hybrid accumulation. Nucleic Acids Res. 2016; 44:718–729. 26527723 10.1093/nar/gkv1152 PMC4737143

[125] Arimondo P.B. , Riou J.-F., Mergny J.-L., Tazi J., Sun J.-S., Garestier T., Hélène C. Interaction of human DNA topoisomerase I with G-quartet structures. Nucleic Acids Res. 2000; 28:4832–4838. 11121473 10.1093/nar/28.24.4832 PMC115246

[126] Marchand C. , Pourquier P., Laco G.S., Jing N., Pommier Y. Interaction of human nuclear topoisomerase I with guanosine quartet-forming and guanosine-rich single-stranded DNA and RNA oligonucleotides. J. Biol. Chem. 2002; 277:8906–8911. 11756434 10.1074/jbc.M106372200

[127] Lotito L. , Russo A., Chillemi G., Bueno S., Cavalieri D., Capranico G. Global transcription regulation by DNA topoisomerase I in exponentially growing Saccharomyces cerevisiae cells: activation of telomere-proximal genes by TOP1 deletion. J. Mol. Biol. 2008; 377:311–322. 18272174 10.1016/j.jmb.2008.01.037

[128] Kang M. , Muller M., Chung I. Telomeric DNA damage by topoisomerase I. A possible mechanism for cell killing by camptothecin. J. Biol. Chem. 2004; 279:12535–12541. 14729676 10.1074/jbc.M309779200

[129] Berroyer A. , Kim N. The functional consequences of eukaryotic topoisomerase 1 interaction with G-Quadruplex DNA. Genes (Basel). 2020; 11:193. 10.3390/genes11020193 PMC7073998 32059547

[130] van Wietmarschen N. , Merzouk S., Halsema N., Spierings D.C.J., Guryev V., Lansdorp P.M. BLM helicase suppresses recombination at G-quadruplex motifs in transcribed genes. Nat. Commun. 2018; 9:271. 29348659 10.1038/s41467-017-02760-1 PMC5773480

[131] Paeschke K. , Capra J.A.A., Zakian V.A.A. DNA replication through G-Quadruplex motifs is promoted by the Saccharomyces cerevisiae Pif1 DNA helicase. Cell. 2011; 145:678–691. 21620135 10.1016/j.cell.2011.04.015 PMC3129610

[132] Paeschke K. , Bochman M.L., Daniela Garcia P., Cejka P., Friedman K.L., Kowalczykowski S.C., Zakian V.A. Pif1 family helicases suppress genome instability at G-quadruplex motifs. Nature. 2013; 497:458–462. 23657261 10.1038/nature12149 PMC3680789

[133] Drosopoulos W.C. , Kosiyatrakul S.T., Schildkraut C.L. BLM helicase facilitates telomere replication during leading strand synthesis of telomeres. J. Cell Biol. 2015; 210:191–208. 26195664 10.1083/jcb.201410061 PMC4508891

[134] Chan Y.A. , Aristizabal M.J., Lu P.Y.T., Luo Z., Hamza A., Kobor M.S., Stirling P.C., Hieter P. Genome-wide profiling of yeast DNA:RNA hybrid prone sites with DRIP-Chip. PLos Genet. 2014; 10:e1004288. 24743342 10.1371/journal.pgen.1004288 PMC3990523

[135] Graf M. , Bonetti D., Lockhart A., Jolivet P., Teixeira M.T., Luke B. Telomere length determines TERRA and R-Loop regulation through the cell cycle. Cell. 2017; 170:72–85. 28666126 10.1016/j.cell.2017.06.006

[136] Arora R. , Lee Y., Wischnewski H., Brun C.M., Schwarz T., Azzalin C.M. RNaseH1 regulates TERRA-telomeric DNA hybrids and telomere maintenance in ALT tumour cells. Nat. Commun. 2014; 5:5220. 25330849 10.1038/ncomms6220 PMC4218956

[137] Nguyen D.T. , Voon H.P.J., Xella B., Scott C., Clynes D., Babbs C., Ayyub H., Kerry J., Sharpe J.A., Sloane‐Stanley J.A. et al. The chromatin remodelling factor ATRX suppresses R‐loops in transcribed telomeric repeats. EMBO Rep. 2017; 18:914–928. 28487353 10.15252/embr.201643078 PMC5452009

[138] Vannier J.-B. , Pavicic-Kaltenbrunner V., Petalcorin M.I.R., Ding H., Boulton S.J. RTEL1 dismantles T loops and counteracts telomeric G4-DNA to maintain telomere integrity. Cell. 2012; 149:795–806. 22579284 10.1016/j.cell.2012.03.030

[139] Takedachi A. , Despras E., Scaglione S., Guérois R., Guervilly J.H., Blin M., Audebert S., Camoin L., Hasanova Z., Schertzer M. et al. SLX4 interacts with RTEL1 to prevent transcription-mediated DNA replication perturbations. Nat. Struct. Mol. Biol. 2020; 27:438–449. 32398829 10.1038/s41594-020-0419-3

[140] Wu W. , Bhowmick R., Vogel I., Özer Ö., Ghisays F., Thakur R.S., Sanchez de Leon E., Richter P.H., Ren L., Petrini J.H. et al. RTEL1 suppresses G-quadruplex-associated R-loops at difficult-to-replicate loci in the human genome. Nat. Struct. Mol. Biol. 2020; 27:424–437. 32398827 10.1038/s41594-020-0408-6

[141] Sarkies P. , Reams C., Simpson L.J., Sale J.E. Epigenetic instability due to defective replication of structured DNA. Mol. Cell. 2010; 40:703–713. 21145480 10.1016/j.molcel.2010.11.009 PMC3145961

[142] Schiavone D. , Jozwiakowski S.K., Romanello M., Guilbaud G., Guilliam T.A., Bailey L.J., Sale J.E., Doherty A.J. PrimPol is required for replicative tolerance of G quadruplexes in vertebrate cells. Mol. Cell. 2016; 61:161–169. 26626482 10.1016/j.molcel.2015.10.038 PMC4712188

[143] Guilbaud G. , Murat P., Recolin B., Campbell B.C., Maiter A., Sale J.E., Balasubramanian S. Local epigenetic reprogramming induced by G-quadruplex ligands. Nat. Chem. 2017; 9:1110–1117. 29064488 10.1038/nchem.2828 PMC5669467

[144] Šviković S. , Crisp A., Tan-Wong S.M., Guilliam T.A., Doherty A.J., Proudfoot N.J., Guilbaud G., Sale J.E. R-loop formation during S phase is restricted by PrimPol-mediated repriming. EMBO J. 2018; 38:e99793. 30478192 10.15252/embj.201899793 PMC6356060

[145] Sollier J. , Cimprich K.A. Breaking bad: R-loops and genome integrity. Trends Cell Biol. 2015; 25:514–522. 26045257 10.1016/j.tcb.2015.05.003 PMC4554970

[146] Hartono S.R. , Korf I.F., Chedin F. GC skew is a conserved property of unmethylated CpG island promoters across vertebrates. Nucleic Acids Res. 2015; 43:9729–9741. 26253743 10.1093/nar/gkv811 PMC4787789

[147] Lee C.-Y. , McNerney C., Ma K., Zhao W., Wang A., Myong S. R-loop induced G-quadruplex in non-template promotes transcription by successive R-loop formation. Nat. Commun. 2020; 11:3392–3406. 32636376 10.1038/s41467-020-17176-7 PMC7341879

[148] Johnson J.E. , Cao K., Ryvkin P., Wang L.-S., Johnson F.B. Altered gene expression in the Werner and Bloom syndromes is associated with sequences having G-quadruplex forming potential. Nucleic Acids Res. 2010; 38:1114–1122. 19966276 10.1093/nar/gkp1103 PMC2831322

[149] Nguyen G.H. , Tang W., Robles A.I., Beyer R.P., Gray L.T., Welsh J.A., Schetter A.J., Kumamoto K., Wang X.W., Hickson I.D. et al. Regulation of gene expression by the BLM helicase correlates with the presence of G-quadruplex DNA motifs. Proc. Natl. Acad. Sci. U.S.A. 2014; 111:9905–9910. 24958861 10.1073/pnas.1404807111 PMC4103369

[150] Tang W. , Robles A.I., Beyer R.P., Gray L.T., Nguyen G.H., Oshima J., Maizels N., Harris C.C., Monnat R.J. The Werner syndrome RECQ helicase targets G4 DNA in human cells to modulate transcription. Hum. Mol. Genet. 2016; 25:2060–2069. 26984941 10.1093/hmg/ddw079 PMC5062591

[151] Marchetti C. , Zyner K.G., Ohnmacht S.A., Robson M., Haider S.M., Morton J.P., Marsico G., Vo T., Laughlin-Toth S., Ahmed A.A. et al. Targeting multiple effector pathways in pancreatic ductal adenocarcinoma with a G-quadruplex-binding small molecule. J. Med. Chem. 2018; 61:2500–2517. 29356532 10.1021/acs.jmedchem.7b01781 PMC5867665

[152] Zorzan E. , Elgendy R., Giantin M., Dacasto M., Sissi C. Whole-transcriptome profiling of canine and human in vitro models exposed to a G-Quadruplex binding small molecule. Sci. Rep. 2018; 8:17107. 30459395 10.1038/s41598-018-35516-y PMC6244004

[153] Beauvarlet J. , Bensadoun P., Darbo E., Labrunie G., Rousseau B., Richard E., Draskovic I., Londono-Vallejo A., Dupuy J.-W., Nath Das R. et al. Modulation of the ATM/autophagy pathway by a G-quadruplex ligand tips the balance between senescence and apoptosis in cancer cells. Nucleic Acids Res. 2019; 47:2739–2756. 30759257 10.1093/nar/gkz095 PMC6451122

[154] Gomez D. , Lemarteleur T., Lacroix L., Mailliet P., Mergny J., Riou J. Telomerase downregulation induced by the G-quadruplex ligand 12459 in A549 cells is mediated by hTERT RNA alternative splicing. Nucleic Acids Res. 2004; 32:371–379. 14729921 10.1093/nar/gkh181 PMC373291

[155] Marcel V. , Tran P., Sagne C., Martel-Planche G., Vaslin L., Teulade-Fichou M.-P., Hall J., Mergny J.-L., Hainaut P., Van Dyck E. G-quadruplex structures in TP53 Intron 3: Role in alternative splicing and in production of p53 mRNA Isoforms. Carcinogenesis. 2011; 32:271–278. 21112961 10.1093/carcin/bgq253

[156] Didiot M.-C. , Tian Z., Schaeffer C., Subramanian M., Mandel J.-L., Moine H. The G-quartet containing FMRP binding site in FMR1 mRNA is a potent exonic splicing enhancer. Nucleic Acids Res. 2008; 36:4902–4912. 18653529 10.1093/nar/gkn472 PMC2528169

[157] Zhang J. , Harvey S.E., Cheng C. A high-throughput screen identifies small molecule modulators of alternative splicing by targeting RNA G-quadruplexes. Nucleic Acids Res. 2019; 47:3667–3679. 30698802 10.1093/nar/gkz036 PMC6468248

[158] Fay M. , Lyons S., Ivanov P. RNA G-quadruplexes in biology: principles and molecular mechanisms. J. Mol. Biol. 2017; 429:2127–2147. 28554731 10.1016/j.jmb.2017.05.017 PMC5603239

[159] Cammas A. , Millevoi S. RNA G-quadruplexes: emerging mechanisms in disease. Nucleic Acids Res. 2016; 45:gkw1280. 10.1093/nar/gkw1280 PMC5389700 28013268

[160] Huppert J. , Bugaut A., Kumari S., Balasubramanian S. G-quadruplexes: the beginning and end of UTRs. Nucleic Acids Res. 2008; 36:6260–6268. 18832370 10.1093/nar/gkn511 PMC2577360

[161] Kamura T. , Katsuda Y., Kitamura Y., Ihara T. G-quadruplexes in mRNA: a key structure for biological function. Biochem. Biophys. Res. Commun. 2020; 526:261–266. 32209257 10.1016/j.bbrc.2020.02.168

[162] Laguerre A. , Hukezalie K., Winckler P., Katranji F., Tan Chanteloup G., Pirrotta M., Perrier-Cornet J.-M., Wong J.M.Y., Monchaud D. Visualization of RNA-Quadruplexes in live cells. J. Am. Chem. Soc. 2015; 137:8521–8525. 26056849 10.1021/jacs.5b03413

[163] Cammas A. , Dubrac A., Morel B., Lamaa A., Touriol C., Teulade-Fichou M.-P., Prats H., Millevoi S. Stabilization of the G-quadruplex at the VEGF IRES represses cap-independent translation. RNA Biol. 2015; 12:320–329. 25826664 10.1080/15476286.2015.1017236 PMC4615567

[164] Schaeffer C. , Bardoni B., Mandel J., Ehresmann B., Ehresmann C., Moine H. The fragile X mental retardation protein binds specifically to its mRNA via a purine quartet motif. EMBO J. 2001; 20:4803–4813. 11532944 10.1093/emboj/20.17.4803 PMC125594

[165] Miglietta G. , Cogoi S., Marinello J., Capranico G., Tikhomirov A.S., Shchekotikhin A., Xodo L.E. RNA G-quadruplexes in kirsten ras (KRAS) oncogene as targets for small molecules inhibiting translation. J. Med. Chem. 2017; 60:9448–9461. 29140695 10.1021/acs.jmedchem.7b00622

[166] Weldon C. , Dacanay J.G., Gokhale V., Boddupally P.V.L., Behm-Ansmant I., Burley G.A., Branlant C., Hurley L.H., Dominguez C., Eperon I.C. Specific G-quadruplex ligands modulate the alternative splicing of Bcl-X. Nucleic Acids Res. 2018; 46:886–896. 29156002 10.1093/nar/gkx1122 PMC5778605

[167] Morris M.J. , Negishi Y., Pazsint C., Schonhoft J.D., Basu S. An RNA G-quadruplex is essential for Cap-independent translation initiation in human VEGF IRES. J. Am. Chem. Soc. 2010; 132:17831–17839. 21105704 10.1021/ja106287x

[168] Kwok C. , Marsico G., Sahakyan A.V., Chambers V.S., Balasubramanian S. rG4-seq reveals widespread formation of G-quadruplex structures in the human transcriptome. Nat. Methods. 2016; 13:841–844. 27571552 10.1038/nmeth.3965

[169] Ding Y. , Tang Y., Kwok C.K., Zhang Y., Bevilacqua P.C., Assmann S.M. In vivo genome-wide profiling of RNA secondary structure reveals novel regulatory features. Nature. 2014; 505:696–700. 24270811 10.1038/nature12756

[170] Bashkirov V.I. , Scherthan H., Solinger J.A., Buerstedde J.-M., Heyer W.-D. A mouse cytoplasmic exoribonuclease (mXRN1p) with preference for G4 tetraplex substrates. J. Cell Biol. 1997; 136:761–773. 9049243 10.1083/jcb.136.4.761 PMC2132493

[171] Beaudoin J.-D. , Perreault J.-P. Exploring mRNA 3′-UTR G-quadruplexes: evidence of roles in both alternative polyadenylation and mRNA shortening. Nucleic Acids Res. 2013; 41:5898–5911. 23609544 10.1093/nar/gkt265 PMC3675481

[172] Yang S.Y. , Lejault P., Chevrier S., Boidot R., Robertson A.G., Wong J.M.Y., Monchaud D. Transcriptome-wide identification of transient RNA G-quadruplexes in human cells. Nat. Commun. 2018; 9:4730. 30413703 10.1038/s41467-018-07224-8 PMC6226477

[173] Wolfe A.L. , Singh K., Zhong Y., Drewe P., Rajasekhar V.K., Sanghvi V.R., Mavrakis K.J., Jiang M., Roderick J.E., Van der Meulen J. et al. RNA G-quadruplexes cause eIF4A-dependent oncogene translation in cancer. Nature. 2014; 513:65–70. 25079319 10.1038/nature13485 PMC4492470

[174] Bradner J.E. , Hnisz D., Young R.A. Transcriptional addiction in cancer. Cell. 2017; 168:629–643. 28187285 10.1016/j.cell.2016.12.013 PMC5308559

[175] Nie Z. , Hu G., Wei G., Cui K., Yamane A., Resch W., Wang R., Green D.R., Tessarollo L., Casellas R. et al. c-Myc is a universal amplifier of expressed genes in lymphocytes and embryonic stem cells. Cell. 2012; 151:79. 10.1016/j.cell.2012.08.033 PMC3471363 23021216

[176] Kerkour A. , Marquevielle J., Ivashchenko S., Yatsunyk L.A., Mergny J.-L., Salgado G.F. High-resolution three-dimensional NMR structure of the KRAS proto-oncogene promoter reveals key features of a G-quadruplex involved in transcriptional regulation. J. Biol. Chem. 2017; 292:8082–8091. 28330874 10.1074/jbc.M117.781906 PMC5427283

