# G-quadruplex–R-loop interactions and the mechanism of anticancer G-quadruplex binders

**DOI:** 10.1093/nar/gkaa1206

**Published:** 2020-12-08

**Authors:** Giulia Miglietta, Marco Russo, Giovanni Capranico

**Affiliations:** Department of Pharmacy and Biotechnology, Alma Mater Studiorum University of Bologna, via Selmi 3, 40126 Bologna, Italy; Department of Pharmacy and Biotechnology, Alma Mater Studiorum University of Bologna, via Selmi 3, 40126 Bologna, Italy; Department of Pharmacy and Biotechnology, Alma Mater Studiorum University of Bologna, via Selmi 3, 40126 Bologna, Italy

*Nucleic Acids Research*, gkaa944, https://doi.org/10.1093/nar/gkaa944

The Authors made two errors in drawing the chemical structures of Figure 2.

**Figure 2.**
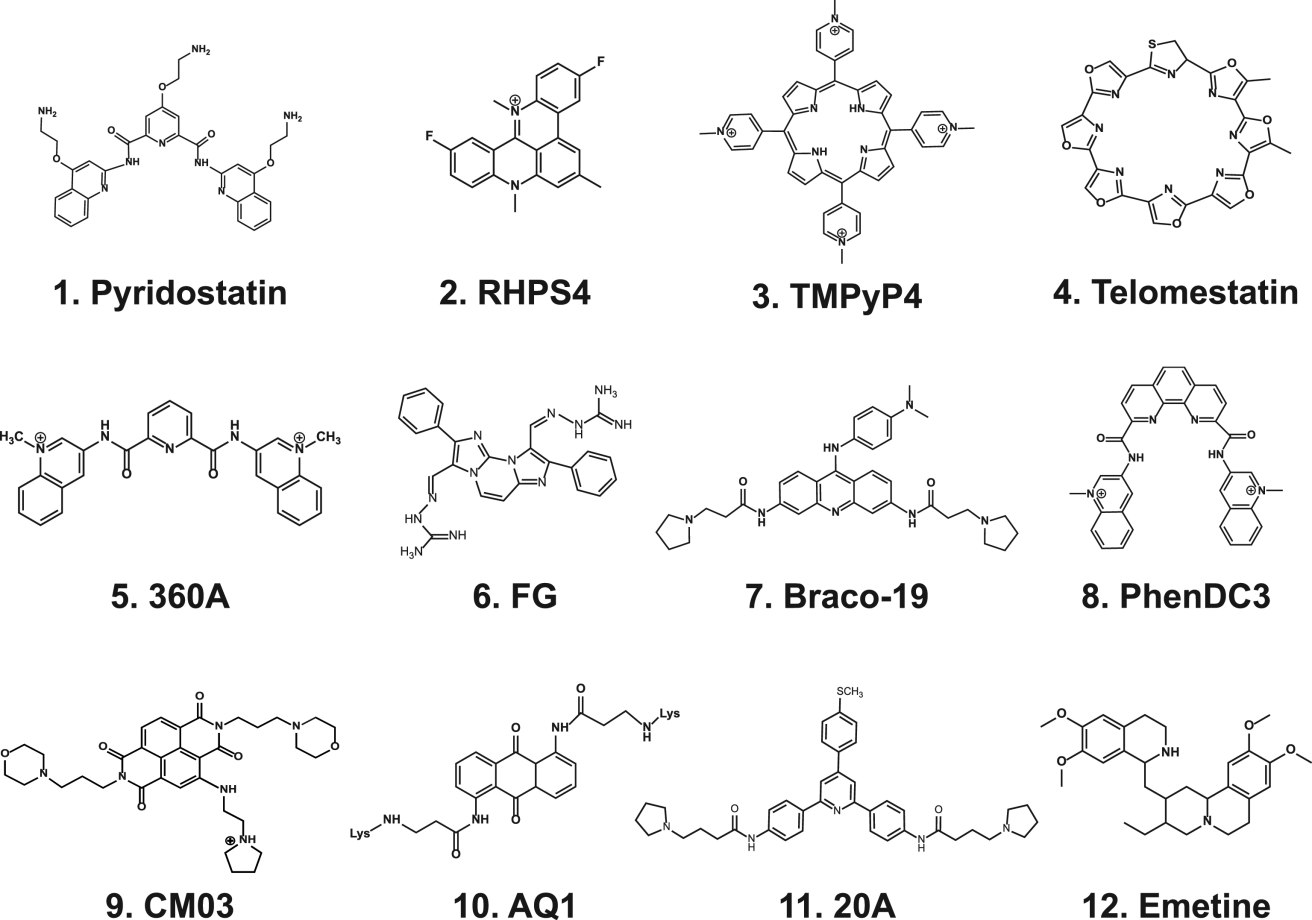
Chemical structures of G4 binders.

1. The structure of compound CM03 lacked a side chain (1-(2-aminoethyl)pyrrolidin-1-ium).

2. The structure of Telomestatin had a Sulphur atom in a wrong ring.

The two errors have been corrected in the new Figure 2. These corrections do not affect the conclusions of the article.

