# Correction to article ‘G-quadruplex–R-loop interactions and the mechanism of anticancer G-quadruplex binders’

**DOI:** 10.1093/nar/gkab483

**Published:** 2021-05-26

**Authors:** Giulia Miglietta, Marco Russo, Giovanni Capranico

**Affiliations:** Department of Pharmacy and Biotechnology, Alma Mater Studiorum University of Bologna, via Selmi 3, 40126 Bologna, Italy; Department of Pharmacy and Biotechnology, Alma Mater Studiorum University of Bologna, via Selmi 3, 40126 Bologna, Italy; Department of Pharmacy and Biotechnology, Alma Mater Studiorum University of Bologna, via Selmi 3, 40126 Bologna, Italy

The authors wish to correct Figure 3 in their article (1). In Figure 3A, the central double arrow has been inverted. The correct figure is shown below.

**Figure 3.**
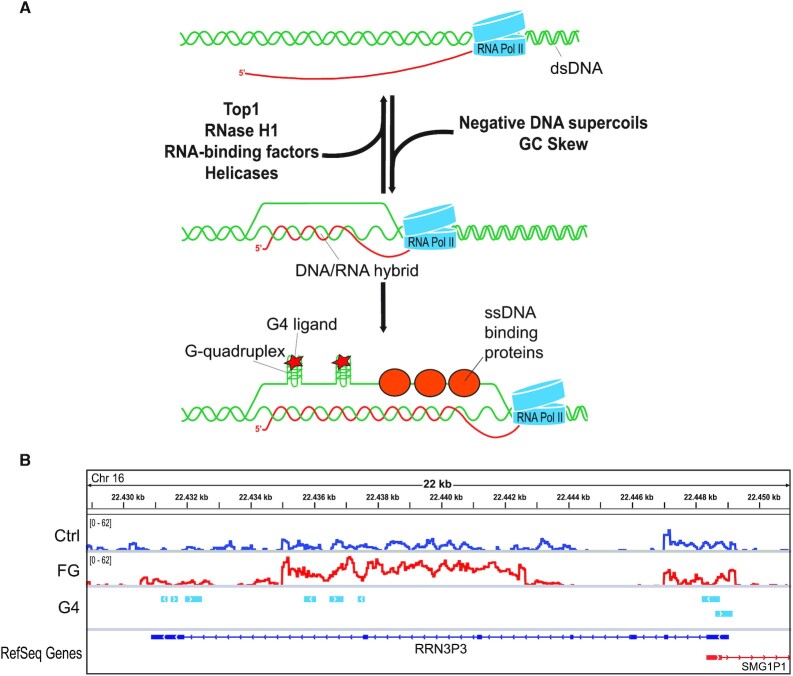
(**A**) Molecular model of the interplay among G4, R-loop, DNA supercoiling and protein factors. Top, main factors that contribute to prevent or promote R loop formation. Below, G4s and factors binding single-strand DNA can stabilize the displaced strand of R-loops. DNA and RNA are shown in green and red, respectively. (**B**) Genomic maps of G4-binder-induced R-loops and PQS established experimentally with a polymerase-stop assay (27) at the RRN3P3 gene locus. The graphs show normalized genomic R-loop profiles for control (blue line) and FG-treated (red line) U2OS cells (71) and oriented PQS (light blue boxes with white arrow).

This change does not affect the results, discussion and conclusions presented in the article. The published article has been updated.
